# Identification of ADAR1i-124: The first effective A-to-I RNA editing inhibitor with promising cancer therapeutic potential

**DOI:** 10.1016/j.isci.2025.114615

**Published:** 2026-01-02

**Authors:** Moeko Minakuchi, Haoran Zhang, Joel Cassel, Yusuke Shiromoto, Jessie Villanueva, Emmanuel Skordalakes, Joseph M. Salvino, Qin Li, Kazuko Nishikura

**Affiliations:** 1 The Wistar Institute, 3601 Spruce Street, Philadelphia, PA 19104, USA; 2 Department of Genetics, School of Medicine, University of Pennsylvania, Philadelphia, PA 19104, USA

**Keywords:** Therapeutics, Enzymology, Nucleic acids, Properties of biomolecules, Cancer

## Abstract

Two ADAR1 isoforms, p150 and p110, are involved in adenosine-to-inosine RNA editing. ADAR1p150-mediated hyper-editing of endogenous dsRNAs prevents their activation of type I interferon signaling-mediated via Melanoma Differentiation-Associated Protein 5 (MDA5), which enables cancer resistance to immune checkpoint blockade. ADAR1p150 also inhibits Z-RNA-mediated activation of Z-DNA Binding Protein 1 (ZBP1) and induction of necroptosis. ADAR1p110 suppresses the formation of telomeric repeat R-loops, which would otherwise induce apoptosis in telomerase-reactivated cancer cells. Together, ADAR1 inhibitors could serve as novel cancer therapeutics. Here, we identified, ADAR1i-124, which inhibits the catalytic activities of both ADAR1p150 and ADAR1p110. ADAR1i-124 activated MDA5 and ZBP1 pathways and dose-dependently inhibited viability across different types of cancer cell lines. Some cancer cell lines, unresponsive to ADAR1i-124 alone, became responsive when co-treated with 5-Aza-CdR. The DNA methylase inhibitor reactivated endogenous retroviruses, leading to the formation of retrovirus dsRNAs and the emergence of a new ADAR1 dependency. Our study establishes the potential of ADAR1i-124 as a future cancer therapeutic.

## Highlights

• Identified ADAR1i-124 as a selective inhibitor of the ADAR1 A-to-I RNA editing activity
• ADAR1i-124 activates the Z-RNA-ZBP1-necroptosis pathway in ADAR1p150^High^ cancer cells
• ADAR1i-124 activates also the dsRNA-MDA5 or -PKR pathway and induces apoptosis
• Combined use of 5-Aza-CdR overcomes resistance to ADAR1i-124 in select cancer cells

## Graphical abstract

**Figure:**
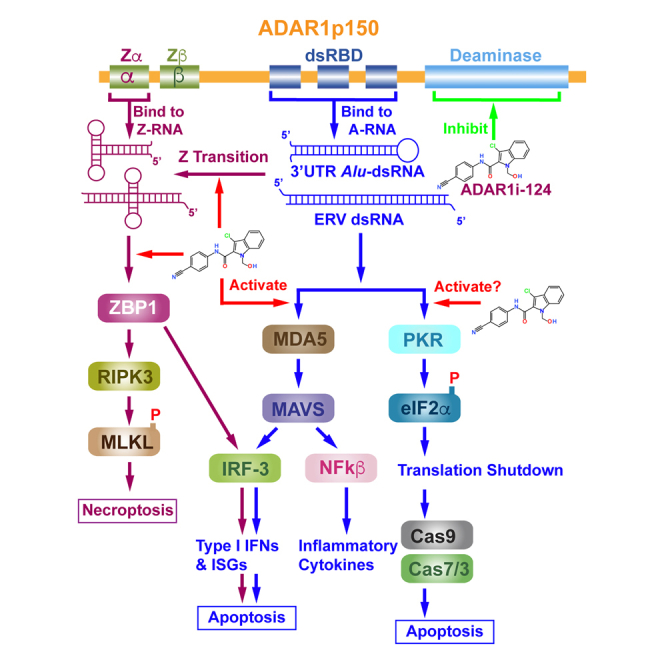

## Introduction

Adenosine deaminase acting on RNA (ADAR) is the enzyme involved in adenosine-to-inosine RNA editing (A-to-I RNA editing).^1^^,^^2^^,^^3^ Three ADAR gene family members (*ADAR1* or *ADAR* or *DRADA*, *ADAR2* or *ADARB1*, and *ADAR3* or *ADARB2*) have been identified in vertebrates.^1^^,^^3^^,^^4^^,^^5^^,^^6^ ADARs share common domain structures, such as multiple dsRNA-binding domains (dsRBDs) and a separate catalytic domain.^7^^,^^8^ A-to-I editing occurs most frequently in non-coding regions that contain the repetitive elements *Alu* and *LINE*,^9^^,^^10^ and many millions of editing sites have been identified in the human transcriptome of these repetitive sequences.^10^^,^^11^^,^^12^ ADAR1 mainly edits repetitive sequences, while ADAR2 targets more frequently protein-coding sequences.^10^^,^^13^ ADAR3 does not have catalytic activity.^14^ ADAR1 and ADAR2 also edit RNA and DNA strands of RNA:DNA hybrid duplexes in addition to dsRNAs.^15^ Although two ADAR1 isoforms, p150 and p110, are both catalytically active enzymes, their cellular localization is different: ADAR1p150 in the cytoplasm and ADAR1p110 in the nucleus.^16^ ADAR1 contains Z-DNA/Z-RNA-binding domains: Zα and Zβ in ADAR1p150 and only Zβ in ADAR1p110.^17^

Despite the remarkable clinical successes of immunotherapy, the development of resistance to the therapy is a major problem. Notably, ADAR1p150 has been identified as one of factors that regulate this resistance to immune checkpoint blockade (ICB).^18^ Regulation of three dsRNA sensors, Melanoma Differentiation-Associated Protein 5 (MDA5), also known as IFIH1 (Interferon Induced with Helicase C Domain 1), Protein Kinase R (PKR or EIF2AK2), and Z-DNA Binding Protein 1 (ZBP1), also known as DAI (Double-Stranded RNA-Activated Inhibitor of Translation), underlies the role of ADAR1p150 in the suppression of ICB responsiveness.^19^ ADAR1p150 interaction with long dsRNAs such as 3′UTR *Alu* dsRNAs and endogenous retrovirus dsRNAs (ERV dsRNAs)^20^ suppresses their activation of dsRNA-sensing mechanisms. First, ADAR1p150 suppresses the MDA5-Mitochondrial Antiviral Signaling Protein (MAVS) pathway, responsible for inducing type I interferon (IFN) signaling and inflammatory responses. ADAR1p150 also suppresses the activation of PKR, responsible for global shutdown of protein synthesis and induction of apoptosis.^19^^,^^21^ ADAR1p150 regulates MDA5 through its A-to-I RNA editing-dependent function. In contrast, the control of PKR by ADAR1p150 may occur through either RNA editing-dependent or RNA editing-independent mechanisms, depending on the specific cell lines involved.^21^^,^^22^ Finally, ADAR1p150 interacts with Z-RNAs via its Zα domain^23^ and suppresses the activation of ZBP1, blocking its induction of necroptosis.^24^^,^^25^^,^^26^^,^^27^^,^^28^ ADAR1p150-mediated A-to-I editing also inhibits the transition of A-form dsRNAs to Z-form, further suppressing ZBP1 activation.^27^^,^^28^

In contrast to functions of ADAR1p150 in dsRNA-sensing mechanisms, ADAR1p110 suppresses the formation of telomeric R-loops specifically in telomerase-reactivated or non-alternative lengthening of telomeres (ALT) cancer cells.^15^ ADAR1p110 edits the RNA strand of telomeric-repeat RNA:DNA hybrids containing mismatched base pairs formed between canonical and variant repeats. This ADAR1p110-mediated editing of mismatched base pairs is required for the degradation of the RNA strands of telomeric-repeat RNA:DNA hybrids by RNase H2. ADAR1p110 editing and consequent suppression of telomeric R-loops is essential for the continued proliferation of telomerase-reactivated cancer cells,^15^ which are in fact 70%–80% of all types of cancers.

These recent discoveries concerning the functions of both ADAR1p150 and ADAR1p110 underscore the pro-oncogenic nature of ADAR1.^15^ We previously identified the chromosomal location of the human ADAR1 gene to 1q21,^29^ a region frequently implicated in gene amplification across various cancer types.^30^^,^^31^^,^^32^^,^^33^^,^^34^ Furthermore, an analysis of the TCGA cancer genome atlas database revealed elevated ADAR1 expression and A-to-I editing levels in almost all types of cancers.^35^ ADAR1 gene amplification and elevated ADAR1 expression levels may contribute to tumor fitness and survival.^34^ Accordingly, ADAR1 inhibitors hold immense promise as effective therapeutics for cancer treatment,^15^ given their potential to disrupt two distinct pro-oncogenic functions of ADAR1: suppression of IFN signaling, resulting in a diminished tumor ICB response (ADAR1p150 function), and the maintenance of telomere stability in telomerase-reactivated cancers (ADAR1p110 function). However, no effective ADAR1 inhibitor is currently available.^36^

To address this gap, we have identified the first effective ADAR1 inhibitor, ADAR1i-124, through high-throughput molecular screening and characterization of its derivatives. ADAR1i-124 demonstrates potent inhibition of ADAR1-mediated A-to-I editing *in vitro* and *in vivo*. ADAR1i-124 activates the dsRNA-sensing pathways, triggers IFN signaling and inflammatory responses, and induces cell death mechanisms across various cancer cell lines. Moreover, combining ADAR1i-124 with an epigenetic inhibitor 5-Aza-2′-deoxycytidine (5-Aza-CdR) enhanced its efficacy, particularly in certain cancer cell lines where the expression of ERV dsRNAs and a new ADAR1 dependency were stimulated. This underscores the potential of ADAR1i-124 for targeted combination therapies. We provide mechanistic insights necessary to validate ADAR1 inhibitors as cancer therapeutics for future clinical utilization.

## Results

### Molecular screening and identification of ADAR1i-124

For molecular screening of ADAR1 inhibitor compounds, we employed a HeLa cell line, stably expressing a dual luciferase reporter system developed by Marie Öhman’s group.^37^ The reporter includes an ADAR1-specific A-to-I editing site, downstream of the firefly luciferase (FFL) cassette, within a short hairpin dsRNA. A-to-I editing changes an amber stop codon to a tryptophan, resulting in a readthrough and in-frame translation of the downstream nano luciferase cassette (Nluc) (Figure S1A). The ratio of Nluc to FFL represents the efficiency of A-to-I editing at the ADAR1-specific^37^ target site. Inhibition of ADAR1 editing activity led to a slow induction of apoptosis in HeLa cells due to telomere instability and mitotic arrest,^15^ which was measured by a reduction in FFL expression (Figure S1B). This ADAR1 depletion-induced apoptosis occurs specifically in non-ALT HeLa cells^15^ (Figure S1C). Based on these findings, we devised a two-step strategy for high-throughput small molecule screening (Figure S1D). The first step involved assessing the induction of “slow” apoptosis on day 3 but not on day 1, while the second step focused on measuring the reduction in Nano Luciferase activity on day 1, which represented the inhibition of A-to-I RNA editing (Figure S1E). The two-step screening of a small molecule library yielded 54 potential candidates (Figure S1E). Using recombinant ADAR1p110 protein and three different substrates, 54 compounds were evaluated by *in vitro* editing assay, which led to the identification of 8 ADAR1 inhibitor compounds (Figure S1F, Table S1). The low hit rate for the identification of true ADAR1 inhibitors may explain the past failures in the identification of ADAR1 inhibitors by molecular screening alone.^37^ We selected ADAR1i-13 (Figure S1F, compound #13) for further studies, as only ADAR1i-13 inhibited ADAR1p110 editing of all the three substrates tested, including the DNA:RNA hybrid substrate (Figure S1F).

**Figure 1:**
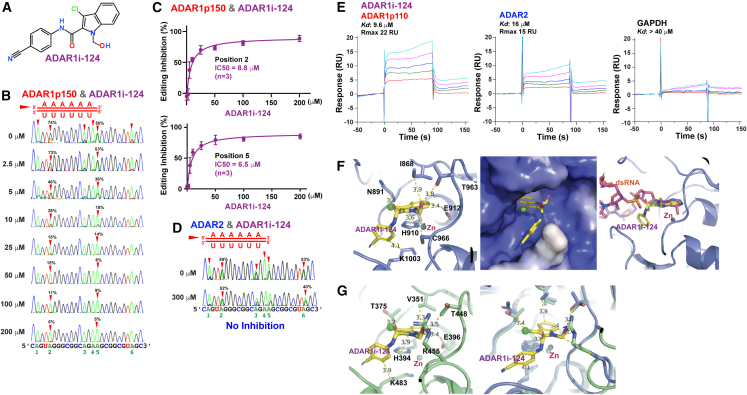
A-to-I RNA editing inhibition and direct binding to ADAR1 by ADAR1i-124 (A) ADAR1i-124 was selected from three compounds with ADAR1 inhibitory activity. (B) *In vitro* editing assay using purified FLAG-ADAR1p150 and a synthetic dsRNA containing six target sites (1–6 position) was conducted in triplicate (*n* = 3) with varying concentrations of ADAR1i-124. The sense RNA strand was analyzed by RT-PCR and cDNA sequencing. The efficiency of A-to-I editing (A-to-G cDNA change) was determined by the guanosine peak (black) replacing the adenosine peak (green) in sequencing chromatograms. (C) Inhibition kinetics analysis resulted in IC_50_ of 8.8 μM and 6.5 μM at position 2 and 5, respectively. Data: mean ± SD (*n* = 3, technical replicates). (D) No editing inhibitory activity was detected for ADAR2 by *in vitro* editing assay conducted at 300 μM. (E) SPR analysis for binding of ADAR1i-124 to ADAR1p110, ADAR2, and a negative control protein GAPDH. (F) Docking simulation of ADAR1i-124 into the catalytic center pocket of a cryo-EM-resolved ADAR1. ADAR1i-124 fits well within the active site near the Zn^2+^ ion, forming close contacts with key residues, including E912 (left). Its nitrile group is nestled within a tight pocket formed by two adjacent loops (middle). Structural alignment with the ADAR1 deaminase domain bound to dsRNA reveals that ADAR1i-124 blocks adenosine access to the active site (right). (G) Docking simulation with the ADAR2 catalytic center shows a conserved binding mode (left). However, structural overlay of ADAR1 (blue) and ADAR2 (green) highlights steric interference from ADAR2 residue R455, which prevents optimal fitting of ADAR1i-124 into the ADAR2 active site (right).

Seven additional analogs of ADAR1i-13 (ADAR1i-120∼126) (Figure S2A, Table S2) were custom-synthesized to elucidate the structural determinants crucial for ADAR1 editing inhibitory activity. *In vitro* editing assay conducted at various concentrations of each analog compound, as an example experiment shown for ADAR1i-124 and ADAR1p150 (Figures 1A and 1B), revealed ADAR1 inhibitory activity of two analogs ADAR1i-120 and ADAR1i-124 (Figure S2B). *In vitro* editing IC_50_ values of ADAR1i-13, ADAR1i-120, and ADAR1i-124 (Figure S3A) were similar, in the ∼10–30 μM range, for both ADAR1p150 (Figures 1C and S3B) and ADAR1p110 (Figure S3C). No substantial ADAR2 inhibitory activity was detected in these three compounds, as shown for ADAR1i-124 examined at 300 μM (Figure 1D). We confirmed no ADAR1 inhibitory activity for 8-azaadenosine and 8-chloroadenosine, claimed previously as ADAR inhibitors,^38^^,^^39^^,^^40^ using *in vitro* editing assay conducted at 300 μM (Figure S3D). The examination of editing inhibition-positive and -negative analogs revealed the significance of the nitrile and chloride substituents for effective A-to-I editing inhibition (Figures S2A and S3A).

### Direct binding of ADAR1i-124 to ADAR1

The catalytic core of ADAR1 houses a zinc ion coordinated by one histidine, H910, and two cysteine residues, C966 and C1036. Additionally, glutamates E912 plays a pivotal role in proton transfer during the hydrolytic deamination reaction.^7^^,^^41^ Surface plasmon resonance (SPR) analysis revealed the direct binding of three editing inhibitors, ADAR1i-13, ADAR1i-120 (Figure S2C), and ADAR1i-124 (Figure 1E), to ADAR1p110. Importantly, ADAR1i-124 did not bind to the negative control protein GAPDH (Figure 1E), and ADAR1 inhibition-negative analogs (ADAR1i-121, -122, −123, −125, and −126) failed to bind to ADAR1p110 (Figure S2C). These results validate the selective binding of ADAR1i-124 to ADAR1, suggesting that its inhibitory action occurs through direct binding to the ADAR1 protein rather than to the dsRNA substrate. Notably, ADAR1i-124 was also found to bind to ADAR2, albeit with a slightly lower affinity (*Kd* = 16 μM and Rmax = 15 RU), compared to its binding to ADAR1 (*Kd* = 9.6 μM and Rmax = 22 RU) (Figure 1E).

Computational docking simulations of ADAR1i-124 using a cryo-EM structure of ADAR1^41^ demonstrate that **ADAR1i-124** binds tightly within the enzyme active site, positioned near the zinc ion and forming close interactions with key residues such as **H910**, **E912**, and **C966**. Additionally, **T963** interacts with the hydroxymethyl group, while **N891** engages with the chloride ion (Figure 1F, left). The nitrile group is buried in a tight pocket within ADAR1, formed by two adjacent loops (residues 1001–1004 and 891–895), likely contributing to the compound’s high binding affinity (Figure 1F, middle). Structural alignment of the ADAR1 deaminase domain bound to dsRNA (**RMSD 0.2 Å**) provides insight into how ADAR1i-124 blocks adenosine access to the active site (Figure 1F, right). A comparable docking simulation using the catalytic center of **ADAR2**^42^ revealed a similar binding pose, maintaining interactions with conserved residues **T375**, **H394**, **E396**, and **T448**, corresponding to **N891**, **H910**, **E912**, and **T963** in ADAR1 (Figure 1G, left). However, **R455** in ADAR2 imposes a steric hindrance that likely disrupts snug accommodation of ADAR1i-124. In contrast, **A970** in ADAR1, the equivalent of ADAR2 R455, does not present this structural clash (Figure 1G, right), potentially explaining the ADAR1 selectivity of ADAR1i-124. However, to fully elucidate the exact binding mode of ADAR1i-124 and its inhibition mechanism, the crystal structure of the ADAR1-ADAR1i-124 complex is required.

### *In vivo* inhibition of ADAR1 editing activity by ADAR1i-124

**Figure 2:**
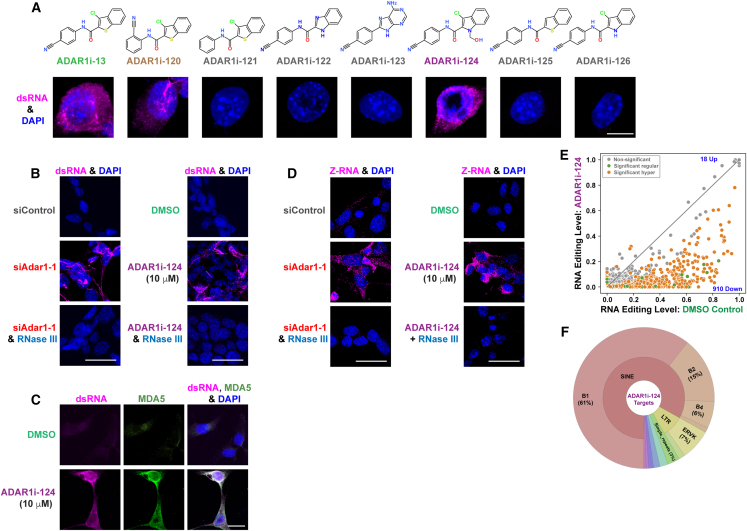
Detection of unedited and/or under-edited dsRNAs and Z-RNAs induced by ADAR1i-124 (A) Immunostaining with the dsRNA-specific mAb J2 revealed unedited and/or under-edited dsRNAs in Yumm1.7 cells, exclusively induced by compounds (10 μM) that inhibit RNA editing. Scale bars, 10 μm. (B) The unedited and/or under-edited dsRNAs induced by siAdar1 and ADAR1i-124 were sensitive to RNase III treatment. Scale bars, 50 μm. (C) Co-localization of unedited and/or under-edited dsRNAs and MDA5 detected by immunostaining using both J2 and anti-MDA5 antibodies. Scale bars, 20 μm. (D) Immunostaining with the Z-RNA-specific mAb Z22 showed that certain dsRNAs underwent a Z-form transition in Yumm1.7 cells treated with siAdar1 and ADAR1i-124. The Z-RNAs induced were also susceptible to RNase III degradation. Scale bars, 50 μm. (E) RNA-seq analysis of Yumm1.7 cells treated with ADAR1i-124 (10 μM 12 h) versus 0.1% DMSO (control) was conducted to assess global A-to-I editing changes. Sites mapped with >20 reads in both samples and >1 edited reads in either sample are plotted. Sites exhibiting significant differences in editing levels (*p* < 0.05, χ^2^ test) between the ADAR1i-124 treatment and DMSO-control are colored in green for regular editing sites and in orange for hyper-edited sites. Sites exhibiting insignificant differences are colored in gray. (F) Types of transposable elements exhibiting significantly lower RNA editing levels upon ADAR1i-124 treatment. Significantly inhibited sites by ADAR1i-124 (*n* = 910) are annotated by RepeatMasker for TE elements.

Suppression of ADAR1 editing activity results in the binding of MDA5 to unedited or under-edited dsRNAs, leading to the oligomerization of MDA5-dsRNA complexes and formation of filament-like structures.^43^ Notably, these unedited and/or under-edited dsRNAs appeared as filament-like structures were detected by immunostaining using J2 mAb specific to unedited dsRNA in mouse Yumm1.7 cutaneous melanoma (CM) cells treated with ADAR1 inhibition-positive compounds, ADAR1i-13, ADAR1i-120, and ADAR1i-124, but were absent in cells treated with ADAR1 inhibition-negative compounds (Figure 2A). Moreover, the unedited and/or under-edited dsRNAs induced by siAdar1 or ADAR1i-124 were effectively abolished by treatment with dsRNA-specific RNase III (Figures 2B, S4A, and S5A). Additional immunostaining using both anti-dsRNA and anti-MDA5 antibodies confirmed the co-localization of unedited and/or under-edited dsRNA with MDA5 within the filament structures (Figure 2C). Furthermore, ADAR1i-124, like siAdar1, prompted the transition of certain dsRNAs from A-form to Z-form, as evidenced by immunostaining using Z22 mAb specific to Z-RNA, in the cytoplasm of Yumm1.7 cells (Figures 2D, S4A, and S5A). The Z-RNA signals were also abolished by RNase III treatment, indicating that they represent Z-dsRNAs but not Z-DNA. These results demonstrate *in vivo* inhibition of ADAR1 editing activity within cells by three compounds with their editing inhibitory activity already confirmed *in vitro* (Figures 1B, 1C, S2B, and S3B).

As expected, RNA-seq analysis showed a global reduction in A-to-I editing *in vivo* in Yumm1.7 cells following treatment with ADAR1i-124 (Figures 2E, S5B, and S6A, and Table S3). A substantial reduction in A-to-I editing levels was demonstrated with three examples of ADAR1i-124 target mRNAs (*Wdr76*, *Iws1*, and *Gnl3l*) (Figure S6B). ADAR1i-124 nearly completely inhibited A-to-I editing at these LTR- and SINE-dsRNA target sites, which are edited up to ∼50%–80%. ADAR1i-124 predominantly targeted SINE transposable elements (TEs), followed by LTRs in Yumm1.7 cells (Figure 2F).

### Suppression of cancer cell viability and induction of IFN signaling by ADAR1i-124

Among the three ADAR1 inhibitors examined, ADAR1i-124 demonstrated the most potent cytotoxicity against HeLa cells, with an IC_50_ of 0.17 μM (Figure S7A). Our prior investigations revealed that ADAR1 knockdown led to the accumulation of R-loops and extensive DNA damage at telomeres, triggering M phase arrest and subsequent apoptosis, specifically in non-ALT cancer cells such as HeLa.^15^ ADAR1i-124 selectively reduced the viability of non-ALT HeLa cells, had a lesser effect on ALT U2OS cells, and showed no impact on IMR90 normal fibroblast cells (Figures S7B and S7C). ADAR1i-124 also induced DNA damage, M phase arrest, and apoptosis (Figures S7D and S5C) along with an increase in R-loop (RNA: DNA hybrid) formation (Figure S7E).

**Figure 3:**
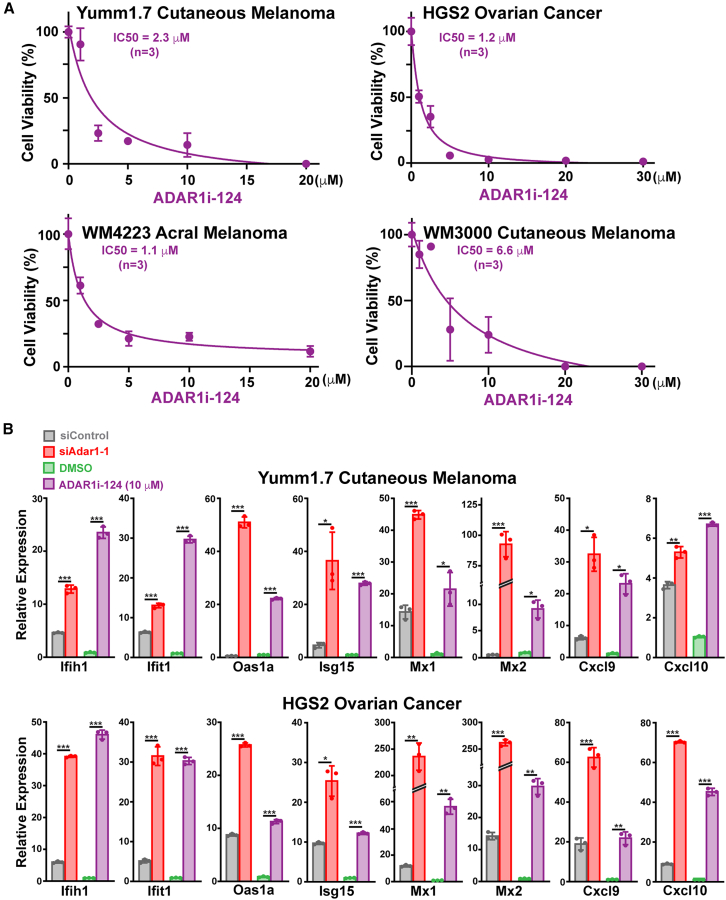
Dose-dependent eradication of cancer cells and activation of IFN signaling (A) Mouse (Yumm1.7 CM, HSG2 OC) and human (WM4223 AM, WM3000 CM) cancer cells were dose-dependently killed by ADAR1i-124 treatment for 48 h. Data: mean ± SD (*n* = 3, biological replicates). (B) Activation of the IFN signaling pathway in Yumm1.7 CM and HGS2 OC cells treated with siAdar1 for 48 h or ADAR1i-124 for 24 h. RNA samples were analyzed by qRT-PCR to monitor ISG expression levels. Data: mean ± SD (*n* = 3, biological replicates). Significant differences were identified by two-tailed Student’s *t* tests: ∗*p* < 0.05; ∗∗*p* < 0.01; ∗∗∗*p* < 0.001.

Cell viability analysis demonstrated that ADAR1i-124 induces dose-dependent cytotoxicity in various mouse and human cancer cell lines (Figure 3A). Finally, ADAR1i-124, like siAdar1, upregulated expression of IFN-stimulated genes (ISGs) such as Ifih1 (MDA5), Cxcl9, and Cxcl10 in Yumm1.7 melanoma and HGS2 ovarian cancer (OC) cells (Figures 3B, S4A, and S5A), indicating that ADAR1i-124 activates IFN signaling, a crucial step for effective ICB.^18^

### Differential ADAR1 dependency developed in cancer cells

**Figure 4:**
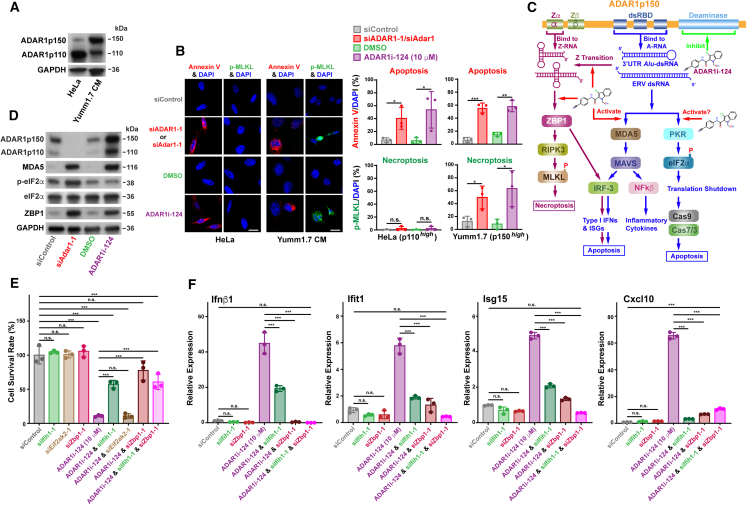
Distinct cell death pathways activated by ADAR1i-124 and mechanisms underlying ADAR1i-124-mediated inhibition of cancer cell viability (A) Differential expression of ADAR1p150 and ADAR1p110 in HeLa and Yumm1.7 cells. Apparent molecular weights (kDa) are indicated. Full blots with molecular weight markers are shown in Figure S5D. (B) Activation of different cell death mechanisms. Annexin V and p-MLKL were used as apoptosis and necroptosis markers, respectively. Scale bars, 20 μm. (left). At least 200 cells were examined for quantitation (right). Apoptosis was predominantly induced in HeLa cells (ADAR1p110^high^), while both apoptosis and necroptosis were activated in Yumm1.7 cells (ADAR1p150^high^) following treatment with siAdar1 or ADAR1i-124. Data: mean ± SD (*n* = 3, biological replicates). Significant differences were identified by two-tailed Student’s *t* tests: *n.s*., not significant; ∗*p* < 0.05. (C) Cytoplasmic ADAR1p150 binds endogenous Z-RNAs and A-form dsRNAs through its Zα domain and dsRNA-binding domain, respectively. Unedited long 3′UTR *Alu* dsRNAs and/or ERV dsRNAs activate dsRNA sensing mechanisms mediated by MDA5 and PKR. A-to-I editing of certain dsRNAs prevents their transition from A-form to Z-form, which may adopt a dumbbell-shaped dsRNA conformation.^24^ ADAR1 regulates the activation of MDA5-MAVS-IFN signaling, as well as PKR activation and the subsequent translation shutdown in response to A-form dsRNAs. ADAR1 also regulates ZBP1-driven necroptosis in response to Z-RNAs. Activation of the Z-RNA-ZBP1 pathway may trigger also apoptosis through IRF3 and RIPK3. ADAR1i-124 activates these ADAR1-regulated pathways by inhibiting the A-to-I RNA editing activity of ADAR1. (D) ADAR1i-124 does not activate the PKR pathway in mouse melanoma cells. Increased expression of ADAR1p150, MDA5, and ZBP1 was detected in Yumm1.7 cells treated with siAdar1 and ADAR1i-124. Phosphorylated eIFf2a, downstream of PKR activation, was detected only in Adar1 knockdown but not in ADAR1i-124 treated Yumm1.7 cells. Apparent molecular weights (kDa) are indicated. Full blots with molecular weight markers are shown in Figure S5E. (E) Inhibition of cell survival rate by ADAR1i-124 in Yumm1.7 cells was rescued by siIfih1 (MDA5) and siZbp1 but not by siEif2ak2 (PKR). (F) ADAR1i-124 induces IFN signaling through the MDA5 and ZBP1 pathways. The activation of IFN/ISG pathways by ADAR1i-124 was fully suppressed by siIfih1 (MDA5) and siZbp1. (E) (F) Data: mean ± SD (*n* = 3 per group, biological replicates). One-way ANOVA followed by Tukey’s post hoc test was used to determine the significance. n.s., not significant; ∗*p* < 0.05; ∗∗*p* < 0.01; ∗∗∗*p* < 0.001.

It has been reported that many human cancer cell lines have developed ADAR1 dependency for continued proliferation.^35^ Gene-Dep analysis^44^ indeed confirmed ADAR1 dependency across different types of cancer cell lines (Figure S8A), whereas no significant ADAR2 dependency was noted (Figure S8B). Notably, ADAR1 knockdown or treatment with ADAR1i-124 activated different cell death mechanisms in ADAR1p110^high^ HeLa and ADAR1p150^high^ Yumm1.7 cancer cells (Figures 4A and S5D). As expected, siADAR1 or ADAR1i-124 exclusively induced apoptosis in HeLa cells (Figures 4B, S4A, and S5A), as detected with Annexin V marker, due to the accumulation of telomeric R-loops as we reported previously.^15^ However, an additional cell death mechanism, necroptosis activated by the ZBP1 pathway, was also induced in Yumm1.7 cells, as detected with the phosphorylated MLKL (p-MLKL) marker (Figures 4B, S4A, and S5A). These findings underscore that ADAR1i-124 elicits distinct cell death pathways (Figure 4C) contingent upon the dominance of either ADAR1p110 or ADAR1p150 in different cancer types. Consequently, ADAR1i-124 emerges as a promising dual-targeting therapeutic strategy, capable of addressing heterogeneous cancer vulnerabilities.

### ADAR1i-124 inhibits only the A-to-I editing activity but not the dsRNA-binding functions of ADAR1

As established, ADAR1 exhibits multiple functions, including A-to-I editing-dependent processes and those reliant on its dsRNA-binding activity, independent of editing.^1^^,^^2^^,^^3^^,^^4^ For example, independently of its A-to-I RNA editing function, ADAR1p110 safeguards mRNAs of SIRT1, a pivotal senescence inhibitor, from HuR-mediated degradation through its dsRNA-binding activity.^45^ Unlike shADAR1, ADAR1i-124 did not induce cellular senescence because it does not interfere with the ADAR1p110 dsRNA-binding activity (Figure S9A). ADAR1 knockout activates MDA5-MAVS-IFN signaling, the Z-RNA-ZBP1 pathway, and also PKR, leading to translation shutdown (Figure 4C).^19^^,^^21^^,^^46^ The activation of the MDA5-MAVS-IFN and Z-RNA-ZBP1 pathways depends on the ADAR1 A-to-I editing function,^22^^,^^24^ whereas the PKR activation has been reported to be A-to-I editing-independent in new born mice and HEK293T human embryonic kidney cells.^22^ This suggests that ADAR1i-124 may be poised to activate the MDA5-MAVS-IFN and Z-RNA-ZBP1 pathways while leaving PKR activation unaffected. Phosphorylated eIF2α (p-eIF2α), downstream of PKR activation, was detected in ADAR1 knockdown but not in ADAR1i-124-treated mouse melanoma cells, indicating no PKR activation exclusively in the latter (Figures 4D, S4A, S5A, and S5E). In contrast, ADAR1i-124, similar to siAdar1, upregulates the expression of two dsRNA sensors, MDA5 and ZBP1. Notably, ADAR1 expression, itself an ISG, was significantly elevated (Figure 4D), suggesting that inhibition of ADAR1-mediated A-to-I editing by ADAR1i-124 triggers a feedback mechanism leading to ADAR1 upregulation.

Rescue experiments using short interfering RNA (siRNAs) targeting downstream components of the ADAR1 pathway (Figure 4C) confirmed that ADAR1i-124-mediated cancer cell eradication depends solely on MDA5 and ZBP1 pathways but is independent of PKR activation in Yumm1.7 mouse melanoma cells used in this experiment (Figures 4E, S4A, S5A, S5F, S5G, S9B, and S9C). Similarly, the pathways involved in ADAR1i-124-mediated IFN and ISG induction were examined. The induction of Ifnβ1 and three ISGs—Ifit1, Isg15, and Cxcl10—triggered by ADAR1i-124 was completely suppressed to basal levels by siIfih1 (MDA5) and/or siZbp1 (Figures 4F, S4A, S5A, S5F, S5G, and S9C). These rescue experiments reaffirm that ADAR1i-124-mediated cancer cell death and IFN/ISG induction are dependent solely on the MDA5 and ZBP1 pathways (Figure 4C), ruling out any non-specific or off-target effects.

### The combined use of ADAR1i-124 and DNMTi reactivates otherwise silenced ERVs, leading to the emergence of ADAR1 dependency

**Figure 5:**
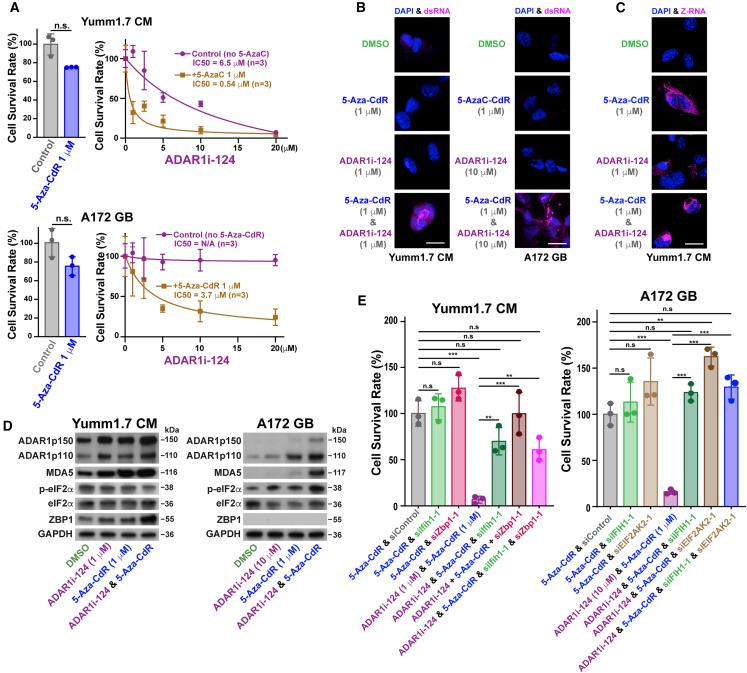
DNA methylation inhibitor 5-Aza-CdR enhances the efficacy of ADAR1i-124 in dose-dependent eradication of certain cancer cell lines less responsive to ADAR1i-124 alone (A) 5-Aza-CdR (1 μM) reduced the IC_50_ of ADAR1i-124 for Yumm1.7 CM cell survival rate by 12-fold. A similar reduction in cell viability was observed with the combination treatment of 5-Aza-CdR in A172 cells, which are completely unresponsive to ADAR1i-124 monotherapy. 5-Aza-CdR (1 μM) alone had no significant effect on the viability of either Yumm1.7 or A172 cells (left graphs). Data: mean ± SD (*n* = 3, biological replicates). Significant differences were identified by two-tailed Student’s *t* tests: n.s., not significant. (B) Immunostaining with J2 antibodies revealed unedited and/or under-edited dsRNAs in Yumm1.7 cells treated with just 1 μM of ADAR1i-124, compared to the previously used 10 μM concentration (see Figure 2B). These unedited and/or under-edited dsRNAs were detected also in A172 cells treated with the combination of ADAR1i-124 (10 μM) and 5-Aza-CdR (1 μM). Scale bars, 20 μm. (C) Immunostaining of Z-RNAs with Z22 antibodies in Yumm1.7 cells treated with 5-Aza-CdR and/or ADAR1i-124 (1 μM). Scale bars, 20 μm. (D) Western blotting analysis for ADAR1 downstream targets in Yumm1.7 and A172 cells treated with ADAR1i-124 and/or 5-Aza-CdR. Apparent molecular weights (kDa) are indicated. Full blots with molecular weight markers are shown in Figure S5H (Yumm1.7) and Figure S5I (A172). (E) The decrease in the Yumm1.7 cell survival rate by a combination of 5-Aza-CdR and ADAR1i-124 was rescued by siIfih1 (MDA5) and siZbp1, while the inhibition of the A172 cell survival rate by a combination of 5-Aza-CdR and ADAR1i-124 was rescued by siIFIH1 (MDA5) and siEIF2ak2 (PKR). Data: mean ± SD (*n* = 3 per group, biological replicates). One-way ANOVA followed by Tukey’s post hoc test was used to determine the significance. n.s., not significant; ∗*p* < 0.05; ∗∗*p* < 0.01; ∗∗∗*p* < 0.001.

Interestingly, recent studies have highlighted the therapeutic potential of DNA-methyltransferase inhibitors (DNMTi) like 5-Aza-2′-deoxycytidine (5-Aza-CdR) and other epigenetic inhibitors in cancer treatment.^20^^,^^47^^,^^48^^,^^49^ For instance, even at low doses, 5-Aza-CdR has been shown to activate previously silenced ERVs, leading to the expression of ERV dsRNAs.^20^^,^^48^^,^^49^ This activation instigates a newfound dependency on ADAR1, even in solid tumors such as melanomas, colon cancers, and OCs.^20^^,^^47^^,^^48^^,^^49^ These discoveries unveil the potential for treating ICB-resistant cancers through a synergistic approach employing ADAR1i-124 and DNMTi or other epigenetic inhibitors. The combination of ADAR1i-124 and 5-Aza-CdR indeed significantly reduced the IC_50_ of ADAR1i-124 by 12-fold in selectively suppressing the survival rate of Yumm1.7 cells (Figure 5A, upper). Furthermore, the combination of ADAR1i-124 and 5-Aza-CdR induced a dose-dependent reduction in the survival rate of human A172 glioblastoma (GB) cells, which were otherwise unresponsive to treatment with ADAR1i-124 alone (Figure 5A, lower). Finally, we found that ADAR1i-124 at 1 μM, rather than 10 μM (Figure 2B), effectively promoted the abundance of unedited and/or under-edited dsRNAs (Figure 5B, left) and upregulated Z-RNA expression (Figure 5C) in Yumm1.7 cells when combined with 1 μM 5-Aza-CdR. Similarly, we confirmed the induction of unedited and/or under-edited dsRNAs in A172 cells treated exclusively with the combination of ADAR1i-124 and 5-Aza-CdR (Figure 5B, right). These findings elucidate the mechanism underlying the synergistic inhibition of cell viability observed in cancer cells subjected to the combination treatment (Figure 5A). Consistent with the results of ADAR1i-124 monotherapy experiments (Figure 4D), no PKR activation (no increased expression of p-eIF2α) was observed in Yumm1.7 cells treated with the combination of ADAR1i-124 and 5-Aza-CdR (Figure 5D, left, and Figure S5H). Notably, the expression levels of the two dsRNA sensors, MDA5 and ZBP1, along with both p150 and p110 isoforms of ADAR1, were upregulated following treatment with either ADAR1i-124 or 5-Aza-CdR alone, and this upregulation was even more pronounced with the combination treatment (Figure 5D, left, and Figure S5H). The findings suggest a markedly increased dependency on ADAR1 and the activation of its two downstream dsRNA sensor-mediated pathways in this mouse melanoma cell line, potentially driven by the enhanced expression of dsRNAs and Z-RNAs induced by 5-Aza-CdR. Indeed, the inhibition of Yumm1.7 cell survival rate by the combination treatment with ADAR1i-124 (1 μM) and 5-Aza-CdR was fully reversed by siIfih1 (MDA5) and siZbp1, confirming that the induced cell death was solely dependent on these two dsRNA sensor pathways (Figure 5E, left, Figures S4A, S5F, S5G, and S9C).

In contrast, the combination treatment in A172 cells upregulated MDA5 and also activated PKR, as indicated by the detection of p-eIF2α (Figure 5D, right, and Figure S5I). Furthermore, the combination treatment led to a significant increase in the expression of ADAR1 (both p150 and p110 isoforms), suggesting a potential new dependency on ADAR1. This may be driven by the substantial induction of ERV dsRNAs (see the following section) caused by 5-Aza-CdR treatment. Interestingly, ZBP1 expression was completely absent in this human GB cell line, even after treatment with ADAR1i-124 and/or 5-Aza-CdR (Figure 5D, right, and Figure S5I). This complete lack of the ZBP1 Z-RNA sensor may be relevant to the unexpected PKR activation in A172 cells, in contrast to no PKR activation in Yumm1.7 melanoma cells. Alternatively, treatment with 5-Aza-CdR may induce a specific class of dsRNAs in this human GB cell line, which activate the PKR pathway unless edited by ADAR1. As expected, the inhibition of A172 cell survival rate was fully rescued by siIFIH1 (MDA5) and, more than notably, by siEIF2AK2 (PKR) (Figure 5E, right, Figures S4B and S5J). These findings suggest that, in this human GB cell line, PKR activation is regulated by the A-to-I RNA editing activity of ADAR1, highlighting the complex nature of the ADAR1-mediated PKR regulation, consistent with previous reports.^21^^,^^22^ Overall, our findings underscore the promising potential of combining ADAR1i-124 with DNA methylation and epigenetic inhibitors for cancers that exhibit reduced sensitivity to ADAR1i-124 monotherapy.

### Identification of the dsRNAs that trigger the activation of IFN signaling and induction of cancer cell death

**Figure 6:**
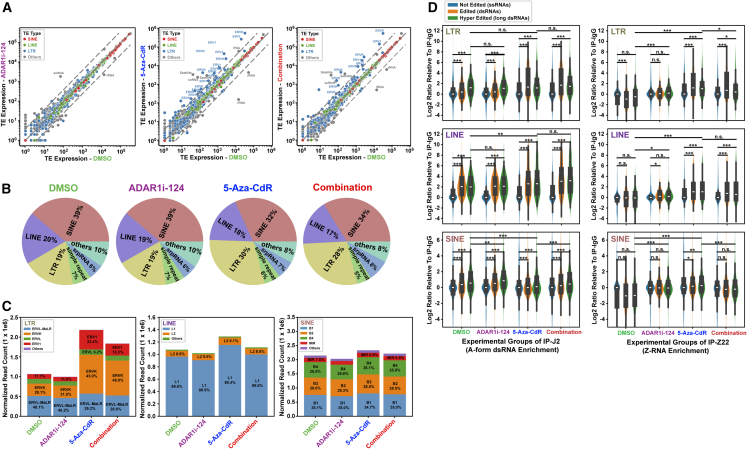
The effects of ADAR1i-124 and 5-Aza-CdR on cellular transposable elements in Yumm1.7 cells (A) Scatterplots comparing the expression level of TEs between DMSO and ADAR1i-124 treatments (left), between DMSO and 5-Aza-CdR treatments (middle), and between DMSO and combination treatments (right). Each point represents a TE subfamily. The dashed lines correspond to fold-changes of 4 and 0.25. (B) Compositions of TEs expressed in DMSO, ADAR1i-124, 5-Aza-CdR, and combination treated samples, irrespective of their dsRNA formation, were analyzed. (C) Stacked bar plots for the LTR, LINE and SINE expressions. The read counts were normalized by the total mapped reads in each sample. Percentages were calculated within each stacked bar. (D) Violin plots showing the IP enrichment of LTR (top row), LINE (middle row), and SINE (bottom row), respectively. J2 (left column) and Z22 (right column) antibodies were used for the IP experiments, respectively (see STAR Methods). The IgG antibody was used as input to calculate the log2ratio of IP enrichment. The edited LTR, LINE, and SINE elements were defined as those containing high-confidence RNA editing sites (coverage >10 and edited reads>1, see STAR Methods). Equal numbers of LTR, LINE, and SINE elements that contain no RNA-editing site (i.e., not edited) were randomly selected from RepeatMasker as control. Hyper-edited TEs are a subset of the edited TEs that have multiple RNA editing sites clustered together within close proximity to each other (see STAR Methods for details). The middle box within each violin indicates the 25%, 50%, and 75% quartiles. Two-way ANOVA and Tukey’s HSD statistical tests were used to determine the significance. n.s., not significant; ∗*p* < 0.05; ∗∗*p* < 0.01; ∗∗∗*p* < 0.001.

ADAR1i-124, either alone (Figures 2B and 2C) or in combination with 5-Aza-CdR (Figure 5B), induced more abundance of unedited and/or under-edited dsRNAs. Additionally, ADAR1i-124 alone (Figure 2D) or in combination with 5-Aza-CdR (Figure 5C) triggered the Z-form transition in certain dsRNAs. To enrich and identify these dsRNAs and Z-form RNAs that activate IFN signaling and the MDA5-and ZBP1-mediated cell death pathways in Yumm1.7 cells (Figure 4C), we conducted RIP RNA-seq analysis on dsRNAs and Z-RNAs immunoprecipitated using the J2 and Z22 monoclonal antibodies, respectively. We confirmed that 5-Aza-CdR treatment significantly increased the expression of LTRs, particularly the ERVK and ERV1 families (Figure 6A). Additionally, LTR, LINE, and SINE together account for ∼80% of total TEs (Figure 6B). The 5-Aza-CdR treatment led to a net increase in all three TEs (Figure 6C). Some of the upregulated TEs are also capable of forming dsRNAs (Figures S10B and 2C, Table S4). These findings suggest that the DNA-methylase inhibitor reactivates endogenous retroviruses, consistent with previous reports.^20^^,^^47^^,^^48^^,^^49^ Furthermore, by comparing hyper-edited TE classes, we found that 5-Aza-CdR treatment alone significantly increased the Z-RNA levels across all three TE classes precipitated by Z22 antibodies (Figure 6D, right), while having a less pronounced effect on the quantity of dsRNAs precipitated by J2 antibodies (Figure 6D, left). The results suggest that 5-Aza-CdR induces a significant amount of dsRNA in the Z-conformation even before the addition of ADAR1i-124, as confirmed by immunostaining experiments (Figure 5C).

In contrast, ADAR1i-124 treatment had little impact on the induction of TE expression (Figures 6A, 6B, and 6C). However, ADAR1i-124 treatment significantly increased the amount of SINE dsRNA precipitated by J2 antibodies (IP_J2) (Figure 6D, lower left) and Z-RNAs precipitated by Z22 antibodies (IP_Z22) across all three TE classes—LTR, LINE, and particularly SINE (Figure 6D, right). The results suggest that ADAR1i-124-mediated inhibition of ADAR1 catalytic activity likely promotes the transition of all three TE dsRNA classes into the Z-conformation. Additionally, the combination treatment with 5-Aza-CdR led to a further increase in A-form SINE dsRNA, as indicated by enhanced precipitation with J2 antibodies (Figure 6D, lower left).

Finally, we analyzed the editing levels of J2-/Z22-enriched TEs (Figures S11A and S11B). Overall, treatment with ADAR1i-124 led to a global reduction in RNA-editing levels (RELs) at sites within J2-/Z22-enriched TEs (including ERVs) compared to DMSO-treated controls. In contrast, the effect on LINEs was minimal, likely due to their inherently low RELs, leaving limited capacity for further inhibition. In the comparison between 5-Aza-CdR and the combination treatment, RELs displayed a mixed pattern (Figures S12A and S12B), likely due to a strong IFN response and feedback-induced upregulation of ADAR1 at later stages (Figure 4D). In the combination group, cells were treated with 5-Aza-CdR for 3 days followed by ADAR1i-124 for an additional 12 h. At this time point, ISGs, including *Adar1*, were upregulated (Figures S12C and S12D), complicating direct comparisons of RELs. Notably, the combination treatment elicited the strongest IFN response, which increased in a dose-dependent manner (Figures S12C and S12D).

These results suggest that ADAR1i-124-mediated inhibition of A-to-I editing increases the fraction of unedited or under-edited A-form immunogenic dsRNAs (Figure 2B), which are recognized by MDA5 (Figure 2C), or the fraction of dsRNAs in the Z-conformation (Figure 2D), which are recognized by ZBP1. The consequent activation of MDA5-and ZBP1-mediated pathways leads to induction of IFN signaling and cell death pathways (Figure 4C). As previously reported,^48^ unedited and/or under-edited dsRNAs (Figures 2B, 2C, and 5B; filament like structures) predominantly consist of SINE dsRNAs, the levels of which are the most strongly affected by ADAR1i-124 (Figures 2F and 6D, lower left), while LTRs and LINEs may serve as additional sources of longer dsRNAs that can bind to the MDA5 sensor when A-to-I editing is inhibited. In summary, the synergistic effects of the ADAR1i-124 and 5-Aza-CdR combination treatment are likely driven by a substantial net increase in the total levels of immunogenic dsRNAs and Z-RNAs across all three TEs.

## Discussion

In 2019, ADAR1 was identified as a key factor regulating cancer resistance to immunotherapy based on ICB.^18^ The activation of the MDA5-MAVS-IFN signaling pathway by endogenous dsRNAs in tumors plays a crucial role in triggering IFNs and inflammatory responses, both of which are essential for enhancing tumor sensitivity to ICB.^18^ Notably, the cytoplasmic ADAR1p150 plays a critical role in this process by hyper-editing these trigger dsRNAs. ADAR1p150-mediated A-to-I editing of endogenous long dsRNA prevents their recognition as “non-self” by dsRNA sensors such as MDA5, PKR, and ZBP1, thereby inhibiting the activation of downstream innate immune responses. Furthermore, A-to-I editing of telomeric-repeat R-loops by the nuclear ADAR1p110 is essential for the sustained proliferation of telomerase-reactivated cancer cells.^15^ These recent findings suggest that ADAR1 inhibitors could serve as promising therapeutics for overcoming cancer and immunotherapy resistance by targeting the pro-oncogenic functions of both ADAR1p150 and ADAR1p110.

Indeed, two adenosine analogs, 8-azaadenosine and 8-chloroadenosine, have been claimed as effective ADAR inhibitors, with several studies reporting their anti-proliferative effects on various cancer cell types.^38^^,^^39^^,^^40^^,^^50^ However, a more recent, rigorous study has shown that these adenosine analogs are not actually ADAR inhibitors,^36^ suggesting that the previously reported anti-cancer activities may stem from their non-specific cytotoxic effects instead. In this study, we confirmed the finding: neither 8-azaadenosine nor 8-chloroadenosine exhibits ADAR1 inhibitory activity, as demonstrated by the *in vitro* editing assay using ADAR1p110 (Figure S3D). Several indirect pharmacological strategies have been proposed, including the elimination of functional ADAR1p150 through the use of the splicing regulator Rebecsinib,^51^ or the inhibition of ADAR1p150 nuclear export to the cytoplasm using KRT-8602, an Xportin 1 inhibitor.^52^ However, the potential off-target effects of these drugs, which could impact the splicing or nuclear export of numerous genes beyond ADAR1, may pose significant risks for patients when used as cancer therapeutics. Thus, no effective ADAR1-specific inhibitors are currently available. Consequently, numerous pharmaceutical companies are actively working toward developing an effective ADAR1 inhibitor.

Notably, a recent paper claims to have identified ZYS-1 as a potential ADAR1 inhibitor.^53^ Surprisingly, the authors assessed the ADAR1 inhibitory activity of this small-molecule compound using a commercially available kit designed to measure mononucleotide inosine levels. The assay system is in fact designed to evaluate the activity of adenosine deaminase (ADA), an enzyme entirely distinct from ADAR. Previously, we and others demonstrated that ADAR cannot catalyze the adenosine deamination reaction, while ADA is incapable of catalyzing the dsRNA-specific A-to-I deamination reaction.^54^^,^^55^ Accordingly, we tested ZYS-1 (Fludarabine-Cl), which has been marketed as an ADAR1 inhibitor, using our *in vitro* editing assay and found that the compound exhibits no inhibitory activity against ADAR1p150, ADAR1p110, or ADAR2 (Figure S3E). Thus, the reported anti-tumor activity of ZYS-1 is unlikely to result from the inhibition of the ADAR1-mediated A-to-I editing mechanism. Our results underscore the necessity of rigorously evaluating candidate inhibitors using robust biochemical and enzymatic assays.

In contrast, our study provides conclusive evidence that ADAR1i-124 effectively inhibits ADAR1 catalytic activity, both *in vitro* and *in vivo*. ADAR1i-124 does not affect ADAR1 dsRNA binding activity, likely because it binds directly to the catalytic center pocket. Therefore, ADAR1i-124 may offer a distinct approach for selectively targeting ADAR1. It is well-established that ADAR1 carries out a range of functions, some of which depend on its A-to-I RNA editing activity, while others rely on its dsRNA-binding ability but are independent of RNA editing.^1^^,^^2^^,^^3^^,^^4^ For example, ADAR1 is known to modulate RNA interference,^56^ inhibit cellular senescence,^45^ and suppress the stress response-mediated mRNA decay,^57^ all of which occur independently of its A-to-I RNA editing activity. Deficits in some of these A-to-I editing-independent functions of ADAR1 likely contribute to the premature death and the organ-specific defects observed in newborn *Adar1*^−/−^:*Ifih1*^−/−^ mice.^22^^,^^58^ These defects are fully rescued in *Adar1*^*E861A/E861A*^:*Ifih1*^−/−^ mice, which retain ADAR1 dsRNA-binding functions.^59^ ADAR1i-124 is unlikely to interfere with the ADAR1 functions underlying these specific defects observed in the mutant mice.

In this regard, of the most relevance to the future use of ADAR1i-124 as a cancer therapeutic would be any activation of PKR. ADAR1i-124 did not induce PKR activation in ADAR1p150^high^ Yumm1.7 mouse melanoma cells, perhaps due to its lack of effect on dsRNA binding by ADAR1. Several strategies can be employed to target ADAR1, including gene inactivation through knockout techniques or direct protein degradation approach (dTAG system).^60^ However, these methods would target both the catalytic and dsRNA-binding activity of ADAR1, leading to the activation of not only MDA5 and ZBP1 pathways and IFN signaling, but also PKR.^21^^,^^22^ In contrast, ADAR1i-124 offers a distinct advantage by selectively activating IFN signaling and triggering MDA5-and ZBP1-mediated cell death pathways, without inducing PKR-driven protein translation shutdown, at least in ADAR1p150^high^ Yumm1.7 cells. Widespread PKR activation and the resulting protein translation shutdown could cause severe side effects in patients treated with a therapy that affects both ADAR1 catalytic and dsRNA-binding functions. Additionally, recent studies suggest that PKR activation impairs antigen-specific CD8 T cell expansion and functions, thereby suppressing anti-tumor immunity.^61^^,^^62^ Given these factors, ADAR1i-124 emerges as a highly promising therapeutic candidate for treating cancer treatment, particularly for patients resistant to ICB. It must be noted that ADAR1i-124 did activate PKR in ADAR1p110^high^ A172 human GB cells. Additional studies are required to understand the mechanism that controls differentially the PKR activation in different cancer cell lines.^21^

Our findings show that ADAR1i-124, when used as a single agent, effectively induces cell death in various cancer cell lines, highlighting its potential as a standalone cancer therapy independent of ICB therapy. Notably, we also discovered that the DNA-methylase inhibitor 5-Aza-CdR significantly enhances the efficacy of ADAR1i-124, restoring its ability to inhibit the viability of certain cancer cell lines that were previously unresponsive to ADAR1i-124 alone. This synergy is probably a result of the *de novo* induction of ERV dsRNAs, primarily derived from ERVK and ERV1 class TEs, by 5-Aza-CdR, thus creating a new or additional ADAR1 dependency in those cancer cells. Inhibition of ADAR1-mediated A-to-I editing by ADAR1i-124 converts those dsRNAs into triggers, with some in A-form and others in Z-form. They are subsequently recognized by dsRNA sensors such as MDA5 and ZBP1, and even PKR in certain cancer cell lines. Our results suggest a promising strategy would be to use ADAR1i-124 in combination with DNMTi or even other epigenetic inhibitors known to reactivate dormant ERVs^20^^,^^48^^,^^49^ for treating cancers that are less responsive to ADAR1 inhibitor monotherapy.

In conclusion, we have identified ADAR1i-124, the first and effective ADAR1 inhibitor, and demonstrated its promise for use as a powerful tool to investigate the multi-faceted functions of ADAR1 and also as the basis for a future therapeutic to treat cancers and immunotherapy resistance.

### Limitations of the study

Our study primarily focused on the discovery of ADAR1i-124 and its thorough *in vitro* evaluation as an ADAR1 inhibitor. Through biochemical and enzymatic analyses, we demonstrated its strong inhibitory effect on the catalytic activities of both ADAR1p150 and ADAR1p110, while preserving its dsRNA-binding ability. Furthermore, we assessed its potential as a cancer therapeutic *in vivo* using various mouse and human cancer cell lines, gaining mechanistic insights into how it targets mainly the Z-RNA-ZBP1 and dsRNA-MDA5 axis pathways. However, our findings regarding its effects on the PKR activation pathway were inconsistent across different cancer cell lines. In mouse melanoma Yumm1.7 cells, ADAR1i-124 did not activate the PKR pathway, likely because it inhibits only ADAR1 catalytic activity without disrupting its dsRNA-binding function. In contrast, treatment with ADAR1i-124 led to PKR activation in human A172 GB cells, suggesting that PKR pathway regulation is influenced by ADAR1 catalytic activity. Further studies are required to elucidate the mechanistic differences underlying PKR activation in different cellular contexts. The next logical step is the preclinical evaluation of ADAR1i-124 *in vivo* using animal tumor models with both mouse and human cancer cell lines in immune-competent hosts. To determine its true potential as a cancer therapeutic or immunotherapy enhancer, ADAR1i-124 should be tested alone or in combination with epigenetic inhibitors such as 5-Aza-CdR and/or anti-PD1 antibodies. Additionally, before moving forward with such *in vivo* studies, efforts will be made to identify more potent inhibitors by testing structural analogs of ADAR1i-124. While these future investigations are necessary, they are beyond the scope of this current study.

## Resource availability

### Lead contact

Further information and request for resources should be directed to the lead contact, Kazuko Nishikura (kazuko@wistar.org).

### Materials availability

ADAR1i-124 can be made available upon request through a collaborative arrangement.

### Data and code availability

• The RNA-seq and RIP RNA-seq data generated during this study have been deposited in the Gene Expression Omnibus (GEO) database under the accession number GSE284082 (https://www.ncbi.nlm.nih.gov/geo/query/acc.cgi?acc=GSE284082). Mouse reference genome GRCm38/mm10, GENCODE release 19 was used to align raw RNA-seq reads and is publicly available (see SnakePipes index: https://zenodo.org/records/4468065).
• Codes for generating the figures of this paper are available on FigShare (https://doi.org/10.6084/m9.figshare.30628541.v1).
• For other items, contact the lead contact upon reasonable request.

## Acknowledgments

We thank Victor Karlström and Marie öhman for RNA editing reporter lentivirus and the cell line transformed with the reporter (HeLa-Nluc-edit) and Medinoah Company, Inc. for their assistance for custom synthesis services. We thank John M. Murray for critical reading of the manuscript. We also thank the Protein Expression, Genomics, and Imaging Shared Facilities of The Wistar Institute’s Ellen and Ronald Caplan Cancer Center, which are supported by the 10.13039/100000054National Cancer Institute (P30 CA010815), for the services provided. The work was supported by grants from the 10.13039/100000002National Institutes of Health (GM040536, CA175058, GM130716, and P50 CA261608 to K.N. and S10OD030245-01 and P30 CA010815-53 to J.M.S.) as well as Emerson Collective Cancer Research Fund and 10.13039/100002224Melanoma Research Foundation to K.N. M.M. was supported in part by fellowships from the 10.13039/100008732Uehara Memorial Foundation and Osamu Hayaishi Memorial Scholarship for Study Abroad.

## Author contributions

M.M., Y.S., J.M.S., Q.L., and K.N. designed the study. M.M., H.Z., J.C., E.S., and Q.L. performed experiments. M.M., H.Z., J.C., E.S., J.M.S., Q.L., and K.N. wrote the manuscript. All authors helped interpret the data and commented on the manuscript.

## Declaration of interests

J.M.S. holds equity in Alliance Discovery, Inc., Barer Institute, and Context Therapeutics and serves as a consultant for Syndeavor Therapeutics, Inc. K.N. and J.M.S. are inventors on patent applications related to ADAR1 small molecule inhibitors and methods of use.

## STAR★Methods

### Key resources table

**Table:** 

REAGENT or RESOURCE	SOURCE	IDENTIFIER
**Antibodies**		

IB: anti-ADAR1	Cho et al.^63^	15.8.6.1
IB: anti-ZBP1	AdipoGen Life Sciences	AG-20B-0010-C100
IB: anti-MDA5	Cell Signaling Technology	5321
IB: anti-PKR	abcam	AB184257
IB: anti-eIF2a	Cell Signaling Technology	5324
IB: anti-Phospho eIF2a	abcam	ab32157
IB: anti-GAPDH	Cell Signaling Technology	2218
IB: anti-gamma H2A.X (phospho S139) antibody	abcam	ab2893
IB: anti-Phospho-Histone H3 (Ser10)	Cell Signaling Technology	3377
IB: anti-Cleaved PARP (Asp214)	Cell Signaling Technology	5625
IB: anti-ADAR2	Cho et al.^63^	1.3.1
IB: anti-DNA-RNA hybrid clone S9.6	MilliporeSigma	MABE1095
IF: anti-Annexin V	Proteintech	11060-1-AP
IF: anti-phospho-MLKL	Cell Signaling Technology	37333
IF: anti-dsRNA (J2)	MilliporeSigma	MABE1134-100UL
IF: anti-Z-DNA (Z22)	absolute antibody	Ab00783-3.3
IF: Goat anti-Mouse IgG (H+L) Cross-Adsorbed Secondary Antibody, Alexa FluorTM 594	Invitrogen	A11005
IF: Goat anti-Rabbit IgG (H+L) Cross-Adsorbed Secondary Antibody, Alexa FluorTM 594	Invitrogen	A11012
RIP: anti-dsRNA (J2)	SCICONS	10010200
RIP: anti-Z-DNA (Z22)	absolute antibody	Ab00783-3.3
RIP: Normal mouse IgG	Santa Cruz Biotechnology	sc-2025

**Experimental models: Cell lines**		

HeLa	ATCC	CCL-2
IMR90	ATCC	CCL-186
U2OS	ATCC	HTB-96
A172	ATCC	CRL-1620
Yumm1.7	ATCC	CRL-3362
Sf9	Thermo Fisher Scientific	11496015
WM3000	Krepler et al.^64^	N/A
WM4223	Krepler et al.^64^	N/A
HGS2	Maniati et al.^65^	N/A

**Oligonucleotides**		

Oligos for *in vitro* editing assay, see Table S5	This paper	N/A
Oligos for quantitative PCR, see Table S5	This paper	N/A
Oligos for siRNA, see Table S5	This paper	N/A

**Software and algorithms**		

GraphPad Prism	GraphPad Software	RRID:SCR_002798
AlphaFold	DeepMind	RRID:SCR_025454
Biacore T200 Evaluation Software	Cytiva	https://www.cytivalifesciences.com/en/us/support/software/biacore-downloads/biacore-t200-software?srsltid=AfmBOorOSA0sZ3RSEoYjT1Znp9DEdDKfyPcr-PirDa1pwWPhgY1fMKtX
CodonCode Aligner	CodonCode Corporation	https://www.codoncode.com/
dbSNP142	UCSC Genome Browser	https://genome.ucsc.edu/
deepTools (v3.5.6)	Ramírez et al.^66^	https://academic.oup.com/nar/article/44/W1/W160/2499308
Image J	NIH	RRID:SCR_003070
ImageQuant	Cytiva	RRID:SCR_014246
IGV (Integrative Genomics Viewer)	Robinson et al.^67^	RRID:SCR_011793
snakePipes (v2.8.0)	Bhardwaj et al.^68^	https://academic.oup.com/bioinformatics/article/35/22/4757/5499080
STAR (v2.7.10b)	Dobin et al.^69^	RRID:SCR_004463
REDIportal	Mansi et al.^70^	RRID:SCR_018490
SPRINT (v0.1.8)	Zhang et al.^71^	https://academic.oup.com/bioinformatics/article/33/22/3538/4004872
vanessa/mpileup (RNA editing level quantification,v1.0)	Vanessa	https://hub.docker.com/r/vanessa/mpileup/
Python (v3.11.11)	Python Software Foundation	https://www.python.org/
seaborn (statistical data visualization, v0.13.2)	Waskom	https://joss.theoj.org/papers/10.21105/joss.03021

**Data and code availability**		

Mouse reference genome GRCm38/mm10, GENCODE release 19, snakePipes index	Zenodo	https://zenodo.org/records/4468065
RNA-seq and RIP RNA-seq data generated in this study (GEO accession number GSE284082)	Gene Expression Omnibus (GEO)	https://www.ncbi.nlm.nih.gov/geo/query/acc.cgi?acc=GSE284082
Python code for generating the figures of this paper	FigShare	https://doi.org/10.6084/m9.figshare.30628541.v1

### Experimental model and study participant details

#### Cell lines

HeLa human ovarian carcinoma (ATCC CCL-2), IMR90 human lung fibroblast (ATCC CCL-186), WM3000 and WM4223 human acral melanoma,^64^ HeLa-Nluc-edit cells,^37^ A172 human glioblastoma,^57^ Yumm1.7 mouse cutaneous melanoma (ATCC CRL-3362), and HGS2 mouse ovarian cancer cell lines^65^ were used in this study. WM3000 and WM4223 cell lines were provided by Dr. Meenhard Herlyn (Wistar). The Yumm1.7 cell line was provided by Dr. Jessie Villanueva (Wistar), while HGS2 cells were collected from HGS2 tumors in C57/B6 mice and provided by Dr. Rugang Zhang at the MD Anderson Cancer Center. HeLa-Nluc-edit cells were generously provided by Dr. Marie öhman. All cell lines were confirmed to be free of mycoplasma contamination. HeLa, IMR90, and A172 cells were cultured in Dulbecco’s Modified Eagle’s Medium (DMEM) (Gibco, 11995-040) supplemented with 10% fetal bovine serum (FBS) (Summerlin Scientific, 100-HI)), penicillin (100 U/ml), and streptomycin (100 mg/ml) (Corning, 30-002-CI). WM3000 and WM4223 cells were cultured in RPMI 1640 (Corning 10-040-CM) supplemented with 5% FBS, penicillin (100 U/ml), and streptomycin (100 mg/ml), while Yumm1.7 cells were cultured in DMEM/F12 (Gibco, 10565-018) supplemented with 10% FBS, penicillin (100 U/ml), and streptomycin (100 mg/ml). HGS2 cells were cultured in DMEM/F12 supplemented with 4% FBS, penicillin (100U/ml), and streptomycin (100 mg/ml), 1% of 100x Insulin (Gibco, 41400045), 0.01 μg/ml of Epidermal Growth Factor (EGF) from murine submaxillary gland (Sigma, E4127), and 1% of 100x GlutaMAX^TM^ Supplement (Thermo, 35050061).

### Method details

#### High-throughput screening assay

HeLa-Nluc-edit cells were seeded at a density of 15,000 cells per well into 384-well white plates (PerkinElmer, 6007689) with 20 μl of DMEM. The plates were incubated overnight at 37°C. Cells were then treated with each compound for 24 hrs, prior to luminescence signal detection. Briefly, to measure Firefly Luciferase activity as an indicator of cell viability, 20 μl of SteadyLite Plus reagents (PerkinElmer, 50-209-9098) were added to each well. The plates were incubated at room temperature for 15 minutes to ensure complete cell lysis and full signal generation. To assess the editing inhibition activity of each compound, 25 μl of Nano-Glo Dual-Luciferase Reporter Assay reagents (Promega, N1610) was added to each well and incubated at room temperature for 10 minutes to allow full signal development. Luminescence signals were measured using CLARIOstar Plus (BMG LABTECH). The ratio between the Nluc and FFL signal was calculated for each well. Results for each plate were then normalized to a negative (0.1% DMSO) and positive control (10 μM Nluc Inhibitor 1^72^). The assay quality was assessed using Z′ factor, calculated with the following formula: Z′ factor = 1 − [(3xSD100%+3x0%)/(Av100%-Av 0%)].^73^

#### Compound screening

A total of 14,400 compounds from the Maybridge HitFinder Screening Library (Maybridge) were used for compound screening. DMSO was used as the negative control, representing 0% inhibition. A DMSO solution containing Nluc Inhibitor 1 (10 μM), representing 100% inhibition, was used as the positive control. The final compound concentration in the screen was 10 μM, with a consistent DMSO concentration of 0.1% in all wells.

#### Synthesis of ADAR1i-124

Detailed synthetic procedures are in Methods S1.

#### Recombinant ADAR protein preparation

All procedures were performed at 4 °C. HAT-ADAR1p110, FLAG-ADAR1p150, and His-ADAR2-expressing Sf9 cells were prepared using baculovirus. The cells were washed with PBS and then resuspended in Tris+ buffer (250 mM Tris pH 7.8, 1 mM dithiothreitol (DTT), 0.6 mM phenylmethylsulfonyl fluoride (PMSF) (Sigma, 329-98-6), and a proteinase inhibitor cocktail (Roche, 11836170001). The cell pellets were sonicated, and the resulting debris was removed by centrifugation. The supernatant (cell extract) was diluted with an equal volume of 2× TGK buffer (100 mM Tris-HCl pH 7.8, 200 mM NaCl, 40% glycerol, 1 mM DTT, 0.6 mM PMSF, and proteinase inhibitor cocktail) and stored at −80 °C. HAT-ADAR1p110 was purified using TALON Metal Affinity Resin (Clontech, PT1320-1). The resin was pre-washed with STD300 buffer (50 mM Tris-HCl pH 7.0, 300 mM NaCl, 20% glycerol, 1 mM β-mercaptoethanol, and 0.05% NP-40). After buffer exchange to STD300 using Zeba 7 K molecular weight cut-off (MWCO) spin desalting column (Thermo Fisher, 89894), the cell extract was loaded onto the resin. After washing with STD300 buffer, the resin was incubated with 80 kU of micrococcal nuclease (NEB, M0247S) for 30 min at room temperature in STD300 buffer containing 1 mM CaCl_2_. The resin was then washed with STD300 buffer containing 0.5 mM EDTA and 0.5 mM EGTA, followed by a wash with STD300 buffer containing 7.5 mM imidazole. HAT-ADAR1p110 recombinant protein was eluted with STD300 buffer containing 150 mM imidazole and the proteinase inhibitor cocktail. Imidazole was removed by using Zeba 7 K MWCO spin desalting column.

#### Preparation of duplex substrates

Sense or antisense oligonucleotides of CDK13 sequences were purchased from IDT and Dharmacon. The 5′ ends of RNA and DNA strands to be analyzed were biotinylated. Sense and antisense oligonucleotides were annealed in annealing buffer (10 mM Tris-HCl, pH 7.5, 50 mM NaCl) to prepare perfectly matched or mismatched dsRNAs, or DNA: RNA hybrids, which were used as substrates in the *in vitro* editing assay.

#### *In vitro* editing assay

The *in vitro* editing reaction mixture, containing 5 nM of CDK13 RNA:RNA duplex substrates and 35 nM of HAT-ADAR1p110, 20 nM of FLAG-ADAR1p150, or 30 nM of His-ADAR2 was incubated at 37 °C for 2 h in *in vitro* editing buffer I (20 mM HEPES-KOH, pH 7.5, 100 mM NaCl, 0.01% NP-40, 5% glycerol, 1 mM DTT). For editing of RNA:DNA hybrid substrates, *in vitro* editing buffer II (20 mM HEPES-KOH, pH 7.5, 20 mM NaCl, 0.01% NP-40, 5% glycerol, 1 mM DTT) was used. Edited RNA or DNA strands were purified using Dynabeads^TM^ MyOne^TM^ Streptavidin C1 (Thermo Fisher Scientific). To remove opposite RNA or DNA strands, RNase H (NEB) or TURBO DNase (Thermo Fisher Scientific, 65001) was used, respectively. For sequencing of edited substrates, reverse transcription PCR (RT-PCR) was performed for RNA strands, while PCR was conducted for DNA strands. Each reaction utilized specific primer sets (Table S5). RT reactions were carried out using SuperScript^TM^ III Reverse Transcriptase (Thermo Fisher Scientific, 18080093), and PCR reactions were performed using Platinum^TM^ Taq DNA Polymerase (Thermo Fisher Scientific, 15966005). PCR products were sequenced using a specific sequencing primer, and the ratio of A and G peaks in the chromatograms were analyzed using CodonCode Aligner (CodonCode Corporation).

#### Surface plasmon resonance (SPR) analysis

Binding of ADAR1i-124 and analog compounds to HAT-ADAR1p110, His-ADAR2, and His-GAPDH was assessed using SPR with the Biacore T200 system. HAT-ADAR1p110 and His-ADAR2 were prepared as described previously^56^ (see below). His-GAPDH was purchased from Abcam (AB223103). Proteins were immobilized on a carboxymethyldextran sensor chip (Xantec Bioanalytics, SCBS-CMD700M) using standard amine coupling procedures with HBS running buffer. Briefly, the chip was washed with 0.1 M sodium borate, pH 9.0, and 1 M NaCl for 3 min at 10 μL/min, followed by activation with freshly prepared 100 mM EDC (1-Ethyl-3-(3-dimethylaminopropyl) carbodiimide (Xantec Bioanalytics, C EDCHCL-1G) and 100 mM NHS (N-Hydroxysuccinimide) (Sigma-Aldrich, 130672) in 50 mM MES buffer, pH 5.0, for 10 min at 10 μl/min. After activation, proteins were captured in 10 mM sodium acetate, pH 5.0, to achieve the desired immobilization level. After a 20 min incubation, the remaining activated sites were blocked with 1M ethanolamine, pH 8.5, for 3 min at 10 μL/min. The running buffer was switched to 10 mM HEPES, pH 7.4, 150 mM NaCl, 5 mM MgCl_2_, 0.5 mM TCEP, 0.005% Tween 20, and 5% DMSO. Compound dilutions were prepared in 100% DMSO at 20X the final concentration, then further diluted into running buffer without DMSO to ensure that the DMSO concentration was the same in both the injection solution and the running buffer. The association time was 1.5 min, the dissociation time 3 min, and the flow rate was set to 30 μl/min. Regeneration cycles were not required. Solvent correction cycles were included to account for the bulk effects of DMSO. Data were analyzed using Biacore Evaluation Software.

#### Docking simulation

Docking of ADR1i-124 was performed with the SwissDock (https://www.swissdock.ch), Autodock Vina software.^74^ ADAR1i-124 was docked into the coordinates of the cryo-EM structure of ADAR1 (PDB ID: 983B)^41^ and the crystal structure of ADAR2 (PDB ID: 5ED1).^42^ The search identified several potential binding sites on the surface of the two proteins with the catalytic center being the top solution. Structural comparison of ADAR1 – ADR1i-124 model with the ADAR2-RNA complex (PDB ID: 5ED1)^42^ clearly shows that binding of ADAR1i-124 to the catalytic center of ADAR1 interferes with adenosine deamination.

#### Preparation of genomic DNA for dot blotting

Cells were treated with siRNA for 72 h and ADAR1i-124 for 48 hrs in a 10 cm dish. After treatment, cells were detached from the dish surface using TrypLE Express Enzyme (Gibco, 12604021) and harvested by centrifugation. After washing with PBS, genomic DNA was purified using the QIAGEN Blood & Cell Culture DNA Midi Kit (QIAGEN, 13323). Briefly, the cell pellet was resuspended in buffer C1. After repeated washes with buffer C1 to remove the cell debris, the nuclear pellet was resuspended in buffer G2 (without RNase A) and treated with 2 mg of proteinase K at 50 °C for 60 min. The nuclear fraction was applied to a QIAGEN Genomic-tip pre-treated with buffer QBT and washed twice with buffer QC. Genomic DNA was eluted with buffer QF and precipitated with 2-propanol. The DNA pellet was washed twice with 80% ethanol and air-dried. Genomic DNA was dissolved in TE buffer (10 mM Tris-HCl, pH 8.0, 1 mM EDTA) and incubated overnight at 4 °C.

#### DNA: RNA hybrid immunoprecipitation

Genomic DNAs (50 μg) were diluted in 250 μl of sonication buffer (10 mM Tris-HCl, pH 8.5, 300 mM NaCl) and sonicated using a Sonicator W-220 (Heat Systems Ultrasonics) 20 cycles at Lv3.5 (10 s ON/40 s OFF). During sonication, the samples were carefully kept on ice to maintain a low temperature. A fraction of the genomic DNA samples (90%, 225 μl) was used for immunoprecipitation with the DNA: RNA Hybrid antibody (S9.6) (Millipore Sigma, MABE1095), while the remaining fractions (10%, 25 μl) was used as the input control. Dynabeads^TM^ Protein A (Invitrogen, 10001D) (100 μl) were blocked with 0.5% BSA in PBS containing 5 mM EDTA overnight at 4 °C. After two washes with DRIP buffer (50 mM Tris-HCl, pH 7.4, 150 mM NaCl, 5 mM EDTA, 1% NP-40, 0.1% sodium deoxycholate), 20 μg of DNA: RNA Hybrid antibody (S9.6) or control mouse IgG (Santa Cruz, sc-2025) was added to the blocked Dynabeads^TM^ in DRIP buffer and incubated overnight at 4 °C. Following two washes with DRIP buffer, the beads were resuspended in 100 μl of DRIP buffer containing 500 U of RNasin Plus inhibitor (Promega, N2611). Sonicated DNA was diluted 1:1 in a buffer containing 100 mM Tris-HCl, pH7.4, 10 mM EDTA, 2% NP-40, and 0.2% sodium deoxycholate. Next, 100 μl of the bead suspension was added to the DNA solution and incubated overnight at 4 °C with rotation. The beads were washed sequentially as follows: 1) twice with DRIP buffer; 2) twice with DRIP high-salt buffer (50mM Tris-HCl, pH7.4, 500 mM NaCl, 5 mM EDTA, 1% NP-40, 0.1% sodium deoxycholate); 3) twice with DRIP Li buffer (50 mM Tris-HCl, pH8.0, 250 mM LiCl, 1 mM EDTA, 0.5% NP-40, 0.5% sodium deoxycholate); 4) once with DRIP TE NaCl+ buffer (100 mM Tris-HCl, pH8.0, 10 mM EDTA, 50 mM NaCl); and 5) once with DRIP TE buffer (100 mM Tris-HCl, pH8.0, 10 mM EDTA). Following the removal of DRIP TE buffer using a magnetic stand, DNA:RNA hybrids were eluted from the beads by incubation in 200 μl of DRIP elution buffer (50 mM Tris-HCl, pH8.0, 10 mM EDTA, 1% SDS) at 65 °C for 30 min with shaking at 1,400 rpm using a MultiTherm shaker (Benchmark Scientific, H5000-H). The beads were separated from the supernatant using a magnetic stand, followed by centrifugation to ensure complete removal. The supernatants were incubated with 80 μg of proteinase K (Roche, 03115828001) and 160 U of RNasin Plus inhibitor (Promega, N2611) at 42 °C for 30 min. DNA:RNA hybrids were purified using the QIAquick Nucleotide Removal Kit (QIAGEN, 13323) and eluted in 200 μl of 5 mM Tris-HCl, pH8.5. For RNase H treatment, 50 μg of sonicated genomic DNAs was preincubated with 25 U of *E. coli* RNase H (NEB, M0297S) overnight at 37 °C.

#### Dot blot analysis of DRIP products

Genomic DNA was diluted in 100 μl of 6×SSC (saline sodium citrate) (CORNING, 46-020-CM) and spotted onto an Amersham Hybond-N+ membrane (Cytiva, RPN203B) using a Bio-Dot Apparatus (Bio-Rad, 1706545). The membranes were cross-linked using ultraviolet (UV) light at 0.24 J/cm^2^, then blocked with 5% non-fat dry milk (LabScientific) in PBS containing 0.1% Tween-20 (PBST) for 1 hr. Subsequently, the membranes were further blocked with SuperBlock buffer (Thermo Fisher Scientific, 37515) for an additional 1 hr at room temperature. The membranes were incubated overnight at 4 °C with anti-DNA:RNA Hybrid antibody (S9.6) (Millipore Sigma, MABE1095) at a concentration of 0.1 μg/ml in SuperBlock buffer. After washing the membranes three times with PBST, they were incubated for 1 hr at room temperature with horseradish peroxidase-conjugated donkey anti-mouse IgG secondary antibody (Jackson ImmunoResearch, 715-035-150) at a concentration of 0.1 μg/ml in SuperBlock buffer. After washing the membranes four times with PBST, dot signals were detected using ECL (GE Healthcare, 12316992) and X-ray films. For RNase H treatment, 1 μg of genomic DNA was preincubated with 2 U of *E. coli* RNase H for 2 h at 37 °C.

#### Cell viability measurement

Cell viability for HeLa, WM3000, and WM4223 cells was measured using the CellTiter-Fluor™ Assay (Promega, G6080) in 96-well plates. The working solution for the CellTiter-Fluor™ Assay was prepared following the manufacturer’s instructions. Briefly, 10 μl of GF-AFC substrate was dissolved in 2 ml of assay buffer. One day prior to the assay, 400 cells were seeded into each well of 96-well plate. One set of plates was treated with compounds, and CellTiter-Fluor™ Assay was performed after 72 hrs incubation. The signal intensity, measured as relative fluorescent units (RFUs), was measured (380 nm/505 nm). Cell viability for Yumm1.7, HGS2, and A172 cells was assessed using trypan blue staining, followed by manual cell counting. For the colony formation assay, siRNA-transfected Yumm1.7 or A172 cells were trypsinized and reseeded into fresh medium. Cell numbers were counted and adjusted to the appropriate concentration for seeding onto 6-well plates (1,000 cells/well), then incubated overnight to allow attachment. Cells were cultured in the presence or absence of ADAR1i-124 for 6-8 days. The growth medium, with or without ADAR1i-124, was replaced every 2 or 3 days. The colonies were fixed with 4% paraformaldehyde and stained with 0.5% crystal violet solution. All experiments were conducted in triplicate, and representative results are presented.

#### Senescence induction and senescence-associated β-gal staining

For positive control of the senescence induction, IMR90 cells were infected with lentivirus encoding shADAR1. IMR90 cells were treated with 10 μM ADAR1i-124 for 48 h and examined for senescence induction using Senescence β-Galactosidase Staining Kit (Cell Signaling Technology, 9860) following manufacturer’s instruction.

#### RNAi gene knockdown

Gene knockdown experiments were performed using RNA interference with Lipofectamine RNAiMax (Life Technologies, 13778075) with a final concentration of 2 nM short interfering RNA (siRNA). All siRNAs used in this study are listed in Table S5.

#### Immunofluorescence staining

Transfection of siRNAs at 2 nM concentration was carried out as described above. After 48 h, cells were fixed with 4% paraformaldehyde (Electron Microscopy Sciences, 15710) and rinsed with Dulbecco’s PBS. Microscopic images were captured using the Leica TCS SP8 X White Light Laser Confocal Microscope with LAS X software (Leica). The system was equipped with a UV 405 nm diode, Argon, DPS3561, and HeNe594 lasers for imaging. Fluorescent images were acquired using a 63× objective lens, with a 1024 × 1024 pixel frame. For multicolor experiments, the following wavelength settings were used: dsRNAs, Z-RNAs, p-MLKL or Annexin V (Ex 594 nm/Em 604–630 nm). Nuclear morphological analysis was performed by staining each cell with 4′, 6-diamidino-2-phenylindole (DAPI).

#### Immunoblot analysis

Cell lysates were prepared in Laemmli SDS-sample buffer (Boston BioProducts Inc) supplemented with benzonase nuclease (Sigma), a complete EDTA-free proteinase inhibitor cocktail (Roche), and PhosStop phosphatase inhibitor cocktail (Roche). The proteins were then separated using 4–20% SDS-polyacrylamide gel electrophoresis (SDS-PAGE) on pre-cast gels (Bio-Rad). Proteins were transferred onto Immobilon-P PVDF membranes (Millipore) and blocked with 1% BSA diluted in 10% BSA (Thermo Fisher Scientific) prepared in TBS-0.05% Tween 20 (TBS-T). The membranes were then incubated overnight at 4 °C with the primary antibodies. Following incubation with the appropriate secondary antibodies, protein bands were visualized using enhanced chemiluminescence (ECL) detection (GE Healthcare) and X-ray films. Antibodies were diluted in 10% Blocker BSA buffer (Thermo Fisher Scientific). Antibodies used in this study are listed in key resources table.

#### RNA extraction and qRT-PCR analysis

Total RNA samples were extracted using Trizol Reagent (Ambion, 15596026). Reverse transcription was performed using 1 μg of total RNA with SuperScript^TM^ III Reverse Transcriptase (Invitrogen, 18080044). The resulting cDNAs were analyzed by qPCR using Power SYBR Green Master Mix (Thermo Fisher Scientific, 4367659) on a QuantStudio 6 Flex system (Applied Biosystems). Each experiment was conducted in quadruplicate. Primers used are listed in Table S5.

#### RNA-seq analysis

RNA sequencing was performed by the Genomics Core at the Wistar Institute. Input RNA was converted into Illumina sequencing libraries using the Illumina Standard Total RNA Prep, Ribo-Zero Plus Ligation (Illumina, 20040525), following the manufacturer’s instructions with the following modifications. Input RNA, along with Illumina RNA UD Indexes Set A Ligation (Illumina, 20091655), were ligated to the resulting cDNA after second-strand synthesis and end-prepping. Libraries were quantified using Agilent TapeStation (Agilent, 067-5592). Libraries were loaded onto an Illumina NovaSeq X Series Sequencer in pair-end mode (150 bp x 150 bp), with sequencing conducted to achieve 30-40 million reads per sample.

#### dsRNA immunoprecipitation

Anti-dsRNA enrichments were performed using a previously published method.^75^ Total RNAs were harvested from cells treated with DMSO (control), ADAR1i-124, siControl, or siAdar1 using TRIzol reagent (Invitrogen) extraction. RNAs were diluted to 500 ng/μl in NET2 Buffer (50 mM Tris-HCl, pH 7.6, 150 mM NaCl, 3 mM MgCl_2_) and divided into immunoprecipitated fractions (IP, 50 μg RNA) or 1% input RNA (0.5 μg RNA). IP samples were treated with 2 units of diluted single-strand specific RNase I (Thermo Scientific, AM2294, 100 U/μl) and 2 μl of TURBO DNase (Invitrogen, AM2238) at 37 °C for 10 min. Afterward, 200 U of RNasin Plus Ribonuclease Inhibitor (Promega, N2611) was added, and the samples were placed on ice. J2 anti-dsRNA mAb (Scicons, 10010500) (5 μg) was added to the IP fraction, bringing the final volume to 200 μl. The mixture was incubated overnight at 4°C with continuous end-over-end rotation. Protein A Dynabeads (Thermo Scientific, 10001D) were washed twice with NET2 buffer and then blocked for 30 min with 1 mg/ml BSA and 10 μg/ml linear acrylamide (Thermo Scientific, J67830XF). Blocked beads were then added to the J2/RNA mixture and incubated with continued rotating for an additional 2 hrs. RNA-bound beads were washed three times with ice-cold NET2 buffer for 5 min each. RNA was then extracted using TRIzol LS. The aqueous-phase from the TRIzol extraction was then purified using the RNA Clean and Concentrator-5 kit (Zymo Research, R1015) and eluted into 20 μl of elution buffer. The input and dsRNA-enriched fractions were analyzed using Bioanalyzer (Agilent), converted into cDNA, and subjected to RT-qPCR to quantify as a fraction of the input RNA, with β-actin used as the reference gene.

#### Z-RNA immunoprecipitation

Immunoprecipitation for Z-RNA was performed using the anti-Z-RNA mAb (Z22) (Absolute Antibody, Ab00783-3.3), following the same protocol as described for dsRNA immunoprecipitation.

#### RIP-RNA-seq analysis

dsRNA- or Z-RNA-enriched RNA was used for RIP-RNA-seq analysis, performed following the same protocol as RNA-seq analysis described above. J2 and Z22 mAbs were used for immunoprecipitation and enrichment for A-form and Z-form dsRNAs, respectively. The IgG antibody was used as control. The edited LTR, LINE, and SINE elements were defined as those containing high-confidence RNA editing sites (coverage>10 and edited reads>1). By applying these criteria, 764 edited LTRs, 434 edited LINEs, and 5198 edited SINEs were found as edited all samples. Equal numbers of LTRs, LINEs, and SINEs that contain no RNA editing site (*i.e.,* not edited) were randomly selected from RepeatMasker as unedited control TEs. Hyper-edited TEs are a subset of the edited TEs, in which the editing site density passed the DBSCAN clustering threshold as described above (≥5 editing sites within 120 bp window). The coverages on these TEs were called and normalized by the total number of mapped reads, and the log2ratios relative to IP-IgG were calculated to measure the enrichment of these TEs.

#### Expression analysis

We used snakePipes (v2.8.0, standard mRNA-seq and noncoding RNA-seq pipelines)^68^ for RNA-seq data analyses. The raw sequencing reads were mapped to genes and transposable elements (TEs) using STAR.^69^ The resulting RPKM-normalized bigwig files were used for plotting the coverage tracks in IGV. The raw counts of TEs were aggregated by repeat name, family, and class, respectively, and normalized by the total number of mapped reads. Finally, the normalized TE counts were compared between DMSO, ADAR1i-124, 5-Aza-CdR, and combination samples.

#### *De novo* identification of RNA editing sites

The editome used in this work was created by combining an existing mm10 editome from REDIportal^70^ with *de novo* identified RNA editing sites (RESs) from all sequencing samples generated by this work. For each sample, we used SPRINT (v0.1.8) toolkit^71^ for identifying novel RESs, and filtered the results by requiring each RES to have a coverage > 20 and edited reads > 2 in at least 2 samples. We then merged the list of novel RESs with the REDIportal mm10 editome, and filtered out known SNPs from dbSNP142,^76^ which resulted in a final mm10 editome containing 136,393 RESs. Hyper-edited regions were determined by a density-based clustering algorithm (DBSCAN, eps=120, min_samples=5) as previously reported.^77^ The editome was also annotated with UCSC mm10 RepeatMasker to identify edited repetitive elements for meta-analyses mentioned above.

#### RNA editing levelREL quantification

The RNA editing level (REL) was defined as the ratio of G reads divided by the sum of A and G reads at each site (Table S3), as described previously.^78^ To collect all edited reads including those hyper edited and thus not mapped regularly,^18^ we combined the regular STAR-aligned bam files from the mRNA-seq pipeline with SPRINT-aligned hyper-edited reads before counting the edited and unedited reads at each RES. Codes for REL quantification^78^ are available at: https://hub.docker.com/r/vanessa/mpileup/. For REL comparisons of two samples, we required each RES to have (1) coverage > 20 in both samples and (2) > 0 edited reads in either sample, and the significance of REL difference was determined by chi-squared test. The significant targets of ADAR1i-124 were defined as RESs that pass the filters in both DMSO and ADAR1i-124-treated samples, with a significantly lower REL in the ADAR1i-124 group than the DMSO control (p-value < 0.05, chi-squared test). For hyper-edited regions, we calculated RNA editing index, which denotes the sum of G reads divided by the sum of A and G reads for all RES in that region, as previously described.^77^

### Quantification and statistical analysis

All experiments were conducted at least twice independently, yielding consistent results. Image quantitation was performed using ImageJ or ImageQuant software (GE Healthcare). Data were analyzed using Microsoft Excel (Microsoft Corporation) and are presented as means ± standard deviation (S.D.) or standard error of the mean (S.E.M.). Two-tailed *t*-tests were performed for comparison of two groups. For comparison of multi groups, one-way ANOVA followed by Tukey’s post hoc test was used to determine the significance. Statistical significance was defined as *P* < 0.05.

## Supplemental information

Document S1. Figures S1–S12 and Method S1

Table S1. Eight ADAR1 inhibitor compounds identified by molecular screening

Table S2. ADAR1i-13 and its analog compounds subjected to further studies

Table S3. Comparison of RNA editing levels: DMSO vs. ADAR1i-124 (A) RNA editing sites. (B) Hyper-edited regions.

Table S4. New dsRNAs induced by 5-Aza-CdR

Table S5. DNA and RNA oligos and siRNAs used in this study
