# Supplementary material for: Identification of ADAR1i-124: The first effective A-to-I RNA editing inhibitor with promising cancer therapeutic potential
Source: iScience. 2026 Jan 2;29(2):114615. doi: 10.1016/j.isci.2025.114615 (PMC12834841; doi:10.1016/j.isci.2025.114615)
Supplement: Document S1. Figures S1–S12 and Method S1 [file mmc1.pdf]

iScience, Volume *29*

Supplemental information

Identification of ADAR1i-124: The first effective A-to-I RNA editing inhibitor with promising cancer therapeutic potential

Moeko Minakuchi, Haoran Zhang, Joel Cassel, Yusuke Shiromoto, Jessie Villanueva, Emmanuel Skordalakes, Joseph M. Salvino, Qin Li, and Kazuko Nishikura

Supplemental Information

Inventory

- Supplemental Figure S1-S12
- Supplemental Methods

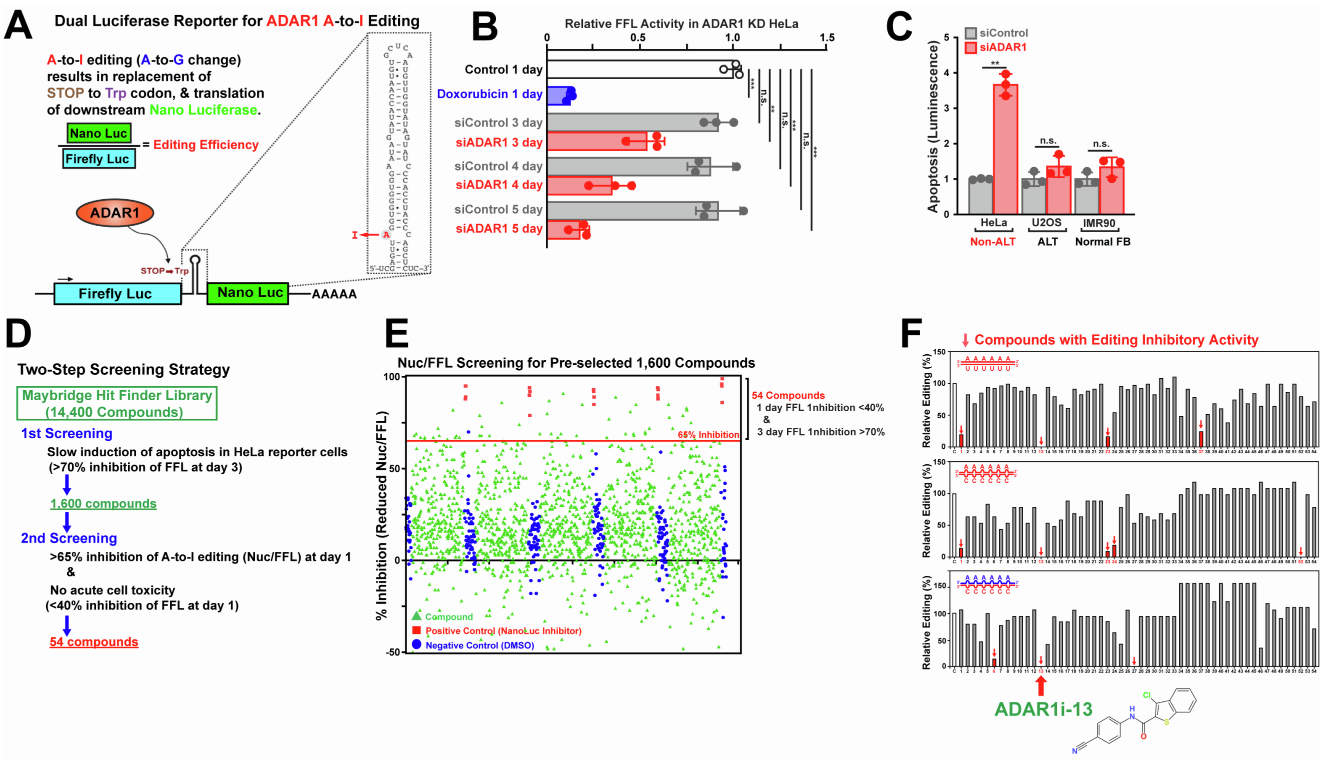

Figure S1. Identification of ADAR1i-13 by molecular screening, related to Figure 1A.

(A) The reporter system specific to ADAR1 mediated A-to-I editing activity[37]. Modified from Fritzell *et al*[37].

(B) Cell viability was measured using FFL activity in HeLa cells. Cytotoxic compounds such as Doxorubicin induce acute apoptosis, which is detected by a decrease in FFL activity. In contrast, ADAR1 depletion leads to the induction of "delayed" cell death 3 to 5 days after treatment.

(C) The delayed apoptosis induced by ADAR1 depletion occurs only in non-ALT (telomerase-reactivated) cancer cells, due to telomere instability and mitotic arrest. Apoptosis was measured using the Apo-Tox Glo system 4 days after siRNA treatment. Significant differences were identified by two-tailed Student's *t*-tests: *$P<0.05$; **$P<0.01$; ***$P<0.001$.

(D) Screening strategy for ADAR1 inhibitors: FFL activity was used to measure cell viability in HeLa cells. Doxorubicin was used as a positive control, inducing acute apoptosis, while ADAR1 depletion or inhibition of its catalytic activity leads to delayed cell death 3 to 5 days after treatment. The first step of the screening pre-selected 1,600 compounds, and the second step identified compounds showing more than a 65% reduction in Nuc expression. Compounds demonstrating acute cytotoxicity (with a >40% reduction in FFL expression on day 1) were excluded.

(E) The 2nd-step screening winnowed the list of candidates down to 54 candidate compounds.

(F) *In vitro* A-to-I editing assay using ADAR1p110 protein and synthetic dsRNA and DNA:RNA hybrid substrates[15] finally identified 8 compounds with ADAR1 inhibitory activity (Table S1).

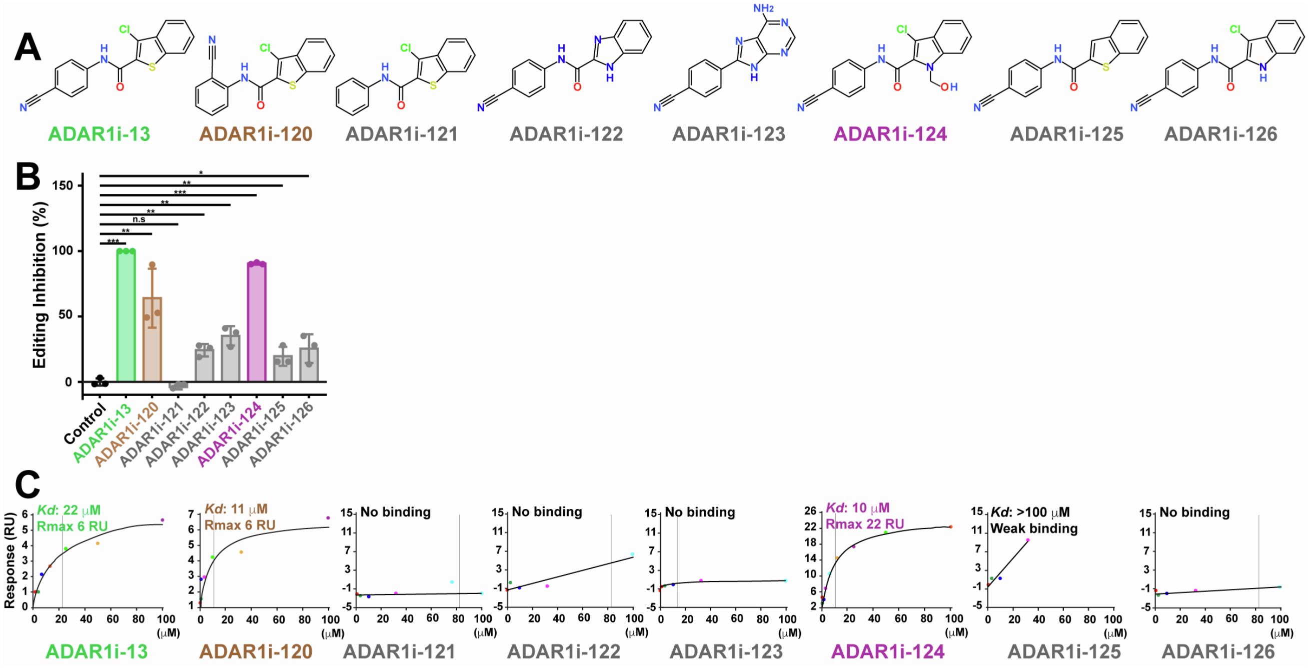

Figure S2. Analysis of ADAR1i-13 analog compounds, related to Figure 1A-C and Figure 2A

(A) Seven additional ADAR1i-13 analog compounds were examined.

(B) *In vitro* editing assay using a synthetic dsRNA substrate conducted at 50 μM of each compound revealed two additional compounds, ADAR1i-120 and ADAR1i-124, which have more than 50% editing inhibitory activity. Data: mean ± SD (n = 3, technical replicates). Significant differences were identified by two-tailed Student's *t*-tests: *n.s*., not significant; **P*<0.05; ***P*<0.01; ****P*<0.001. **c**, SPR analyses for ADAR1p110 binding kinetics of 8 compounds; ADAR1 inhibition positive (ADAR1i-13, -120, and -124) and negative (ADAR1-121, -122, -123, -125, and -126), are shown.

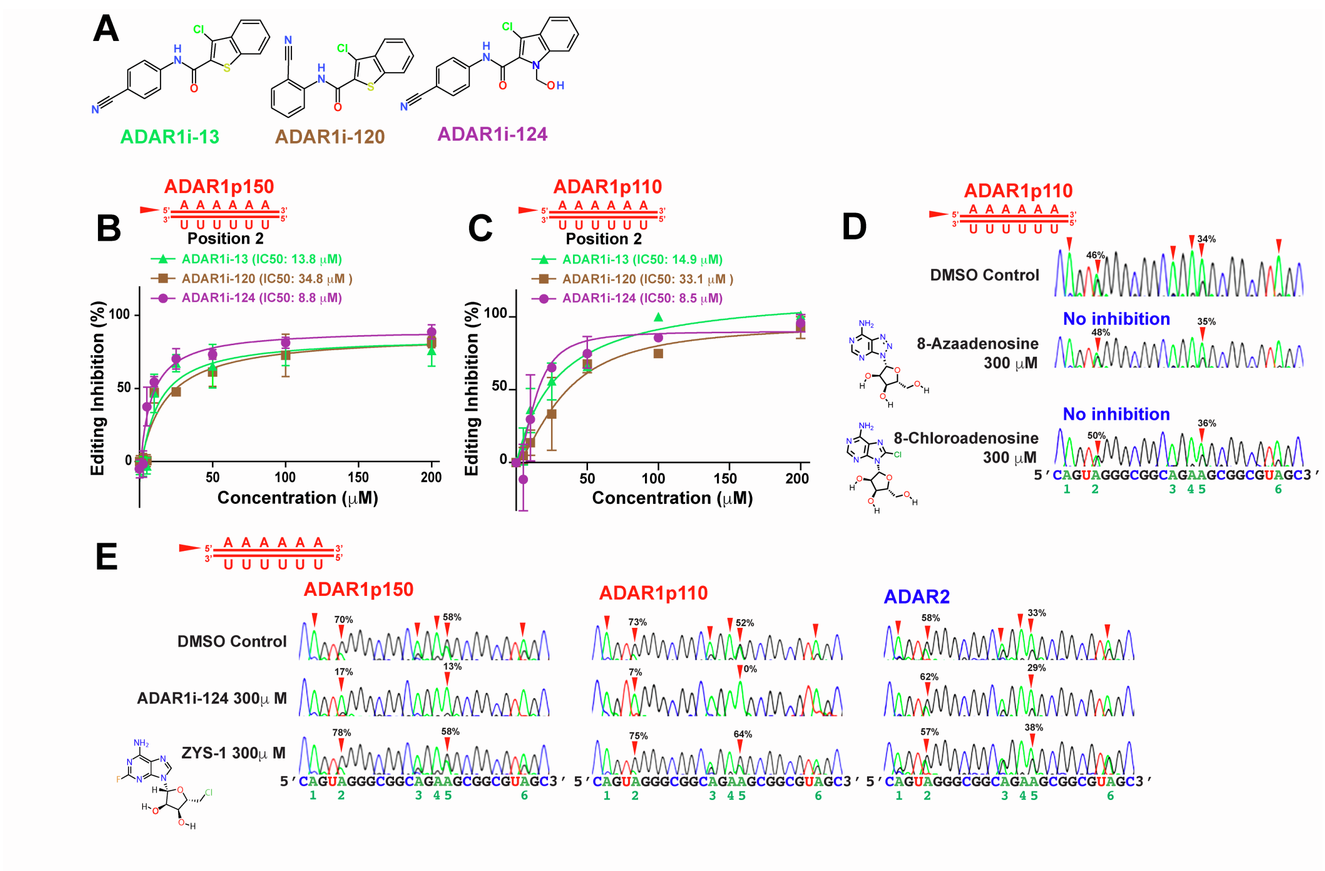

Figure S3. Inhibition of both ADAR1p150 and ADAR1p110 by three ADAR1 inhibitors identified, related to Figure 1A, Figure 2A

(A) Chemical structures of three ADAR1 inhibitor compounds identified. Note the presence of chloride and nitrile groups in all three inhibition-positive compounds.

(B) (C) *In vitro* editing assays using a synthetic dsRNA substrate and ADAR1p150 or ADAR1p110 were conducted with three ADAR1 inhibitors. Data: mean $\pm$ SD (n = 3, technical replicates).

(D) *In vitro* editing assays using a synthetic dsRNA substrate and ADAR1p110 were carried out for 8-Azaadenosine or 8-Chloroadenosine at 300 μM.

(E) *In vitro* editing assays using a synthetic dsRNA substrate were performed with ADAR1p150, ADAR1p110, and ADAR2 in the presence of 300 μM ADAR1i-124 or ZYS-1. ZYS-1 showed no inhibitory activity against any of the ADAR enzymes tested. In contrast, ADAR1i-124 inhibited the activity of ADAR1p150 and ADAR1p110, but not ADAR2.

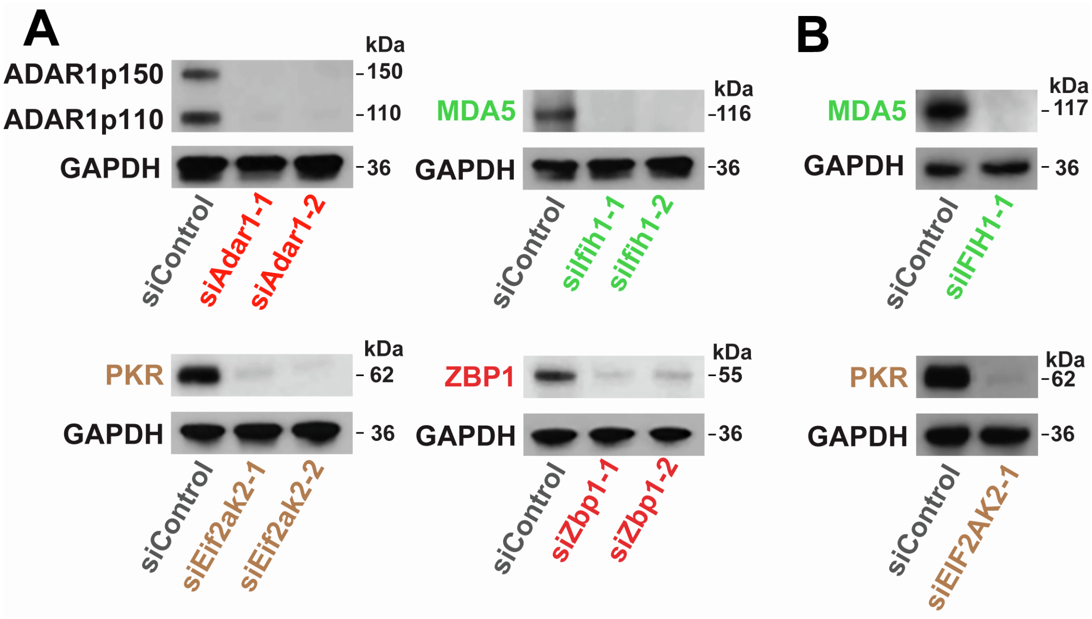

Figure S4. Validation of siRNA gene knockdown, related to Figure 2B, 2C, 3B, 4B, 4D, 4E-F and 5E

(A) The efficacy of mouse siRNAs used for gene knockdown experiments conducted with mouse cells was confirmed by immunoblotting. Apparent molecular weights (kDa) are indicated. Full blots with molecular weight markers are shown in Figure S5B.

(B) The efficacy of human siRNAs used for gene knockdown experiments conducted with human cells was confirmed by immunoblotting. Apparent molecular weights (kDa) are indicated. Full blots with molecular weight markers are shown in Figure S5C.

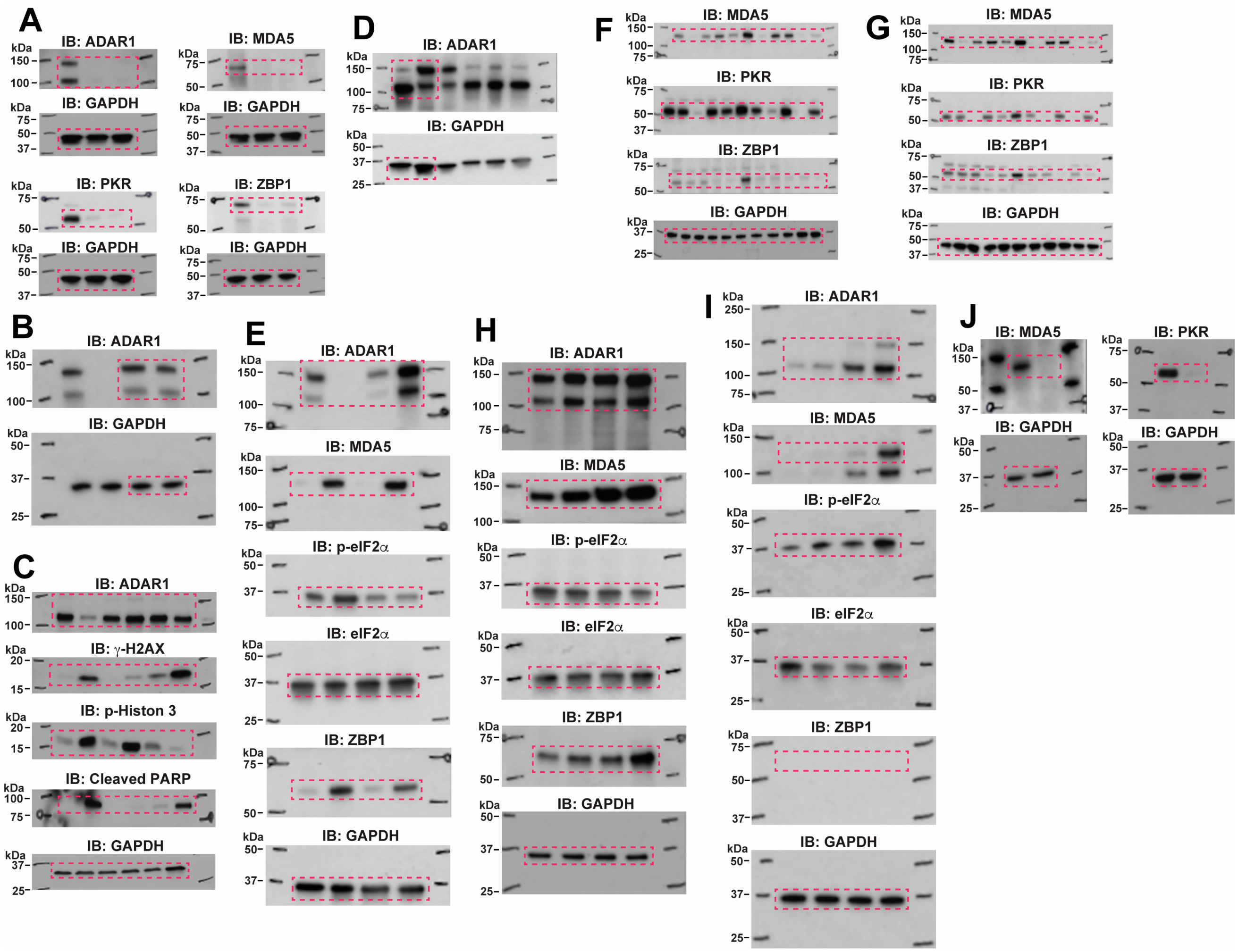

Figure S5. Full immunoblot images with molecular weight markers, related to Figure S4A, S6A, S7D, 4A, 4D, S9C, and 5D

(A) Full immunoblots used for Figure S4A: mouse siRNA set are shown. The cropped areas are indicated by red dashed boxes.

(B) Full immunoblots used for Figure S6A are shown. The cropped areas are indicated by red dashed boxes.

(C) Full immunoblots used for Figure S7D are shown. The cropped areas are indicated by red dashed boxes.

(D) Full immunoblots used for Figure 4A are shown. The cropped areas are indicated by red dashed boxes.

(E) Full immunoblots used for Figure 4D are shown. The cropped areas are indicated by red dashed boxes.

(F) Full immunoblots used for Figure S9C: siRNA-1 set panel are shown. The cropped areas are indicated by red dashed boxes.

(G) Full immunoblots used for Figure S9C: siRNA-2 set panel are shown. The cropped areas are indicated by red dashed boxes.

(H) Full immunoblots used for Figure 5D: Yumm1.7 panel are shown. The cropped areas are indicated by red dashed boxes.

(I) Full immunoblots used for Figure 5D: A172 panel are shown. The cropped areas are indicated by red dashed boxes.

(J) Full immunoblots used for Figure S4B: human siRNA set are shown. The cropped areas are indicated by red dashed boxes.
The molecular weight markers used were Bio-Rad Precision Plus Protein Dual Color Standards.

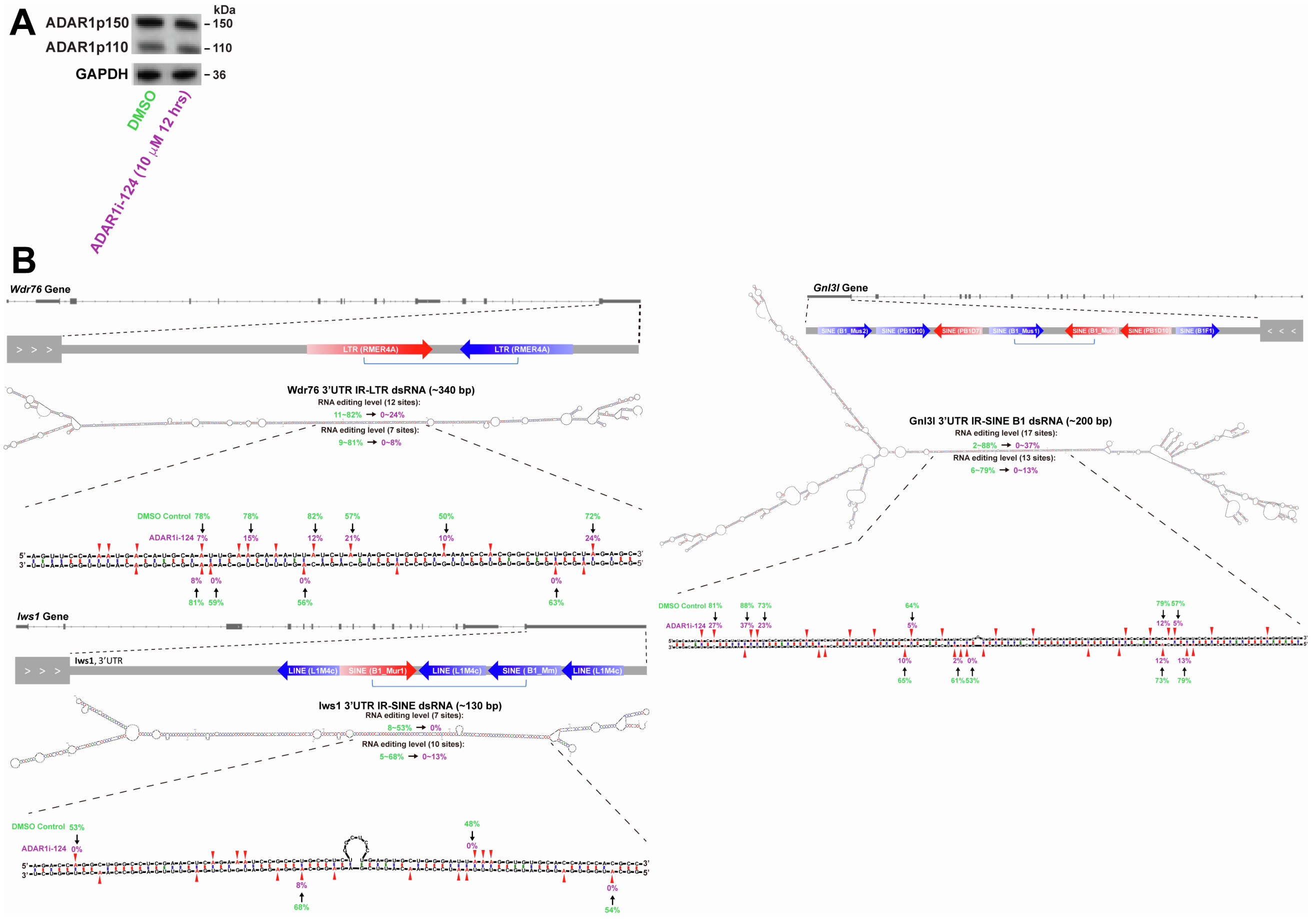

Figure S6. RNA-seq sample preparation and three examples of ADAR1i-124 hyper-editing site targets identified by RNA-seq analysis, related to Figure 2E-F

(A) ADAR1 protein (p150 and p110) expression levels for conditions used for preparation of RNA-seq analysis samples were examined by immunoblotting: Note that no changes in the ADAR1p150 and ADAR1p110 protein levels were noted in Yumm1.7 cells treated with ADAR1i-124 10μM for 12 hrs. Apparent molecular weights (kDa) are indicated. Full blots with molecular weight markers are shown in Figure S5G.

(B) A part of gene organizations, 3'UTR IR-LTR or IR-SINE dsRNA structures predicted by Mfold, and hyper-editing sites identified (red arrow heads) as well as major editing level changes in DMSO control (green) *vs* ADAR1i-124 treatment (purple) are shown. Repetitive elements inserted in the same direction as the host genes are shown in red, and opposite direction in blue. *Wdr76* gene 3'UTR, IR-LTR dsRNA, chr2:121543545-121544544 (+); *IWs1* gene 3'UTR, IR-SINE dsRNA, chr18:32102442-32103041 (+); *Gnl3l* gene 3'UTR IR-SINE dsRNA, chrX:150983730-150985799 (-).

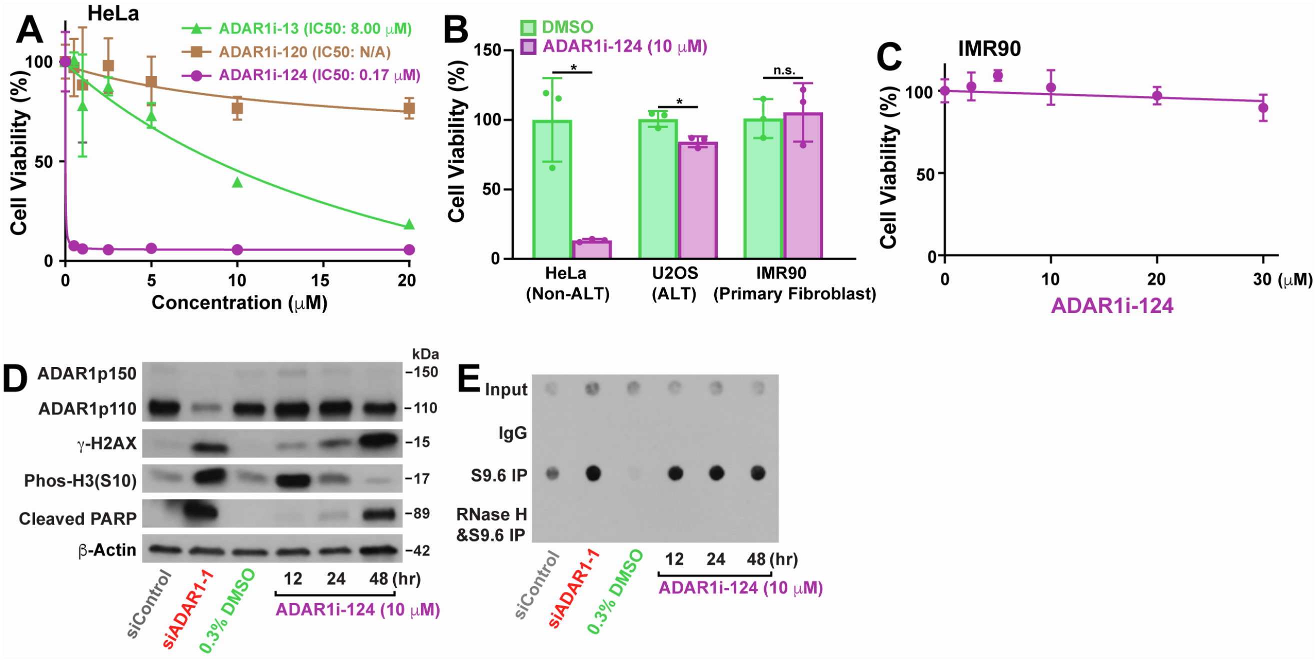

Figure S7. Analysis of ADAR1i-124 effects in cancer cells, related to Figure 3A

(A) More efficient and dose-dependent suppression of HeLa cell viability by ADAR1i-124 than ADAR1i-13 and ADAR1i-120. Data: mean $\pm$ SD (n = 3, biological replicates).

(B) ADAR1i-124 (10 μM) selectively reduced viability of non-ALT HeLa cells. Viability was examined using the Apo Tox-Glo assay system. Control, 0.1% DMSO. Data: mean $\pm$ SD (n = 3, biological replicates). Significant differences identified by two-tailed Student's *t*-tests: *n.s.*, not significant; *P<0.05.

(C) ADAR1i-124 does not significantly affect the viability of normal human fibroblast IMR90 cells.

(D) Western blotting of HeLa cells treated with siADAR1 or ADAR1i-124 (10 μM). Note upregulated γ-H2AX, phosphorylated H3, and cleaved PARP bands. Apparent molecular weights (kDa) are indicated. Full blots with molecular weight markers are shown in Figure S7H.

(E) Dot blot analysis using M Ab S9.6 revealed induction of RNA:DNA hybrids in HeLa cells by siADAR1 or ADAR1i-124 (10 μM).

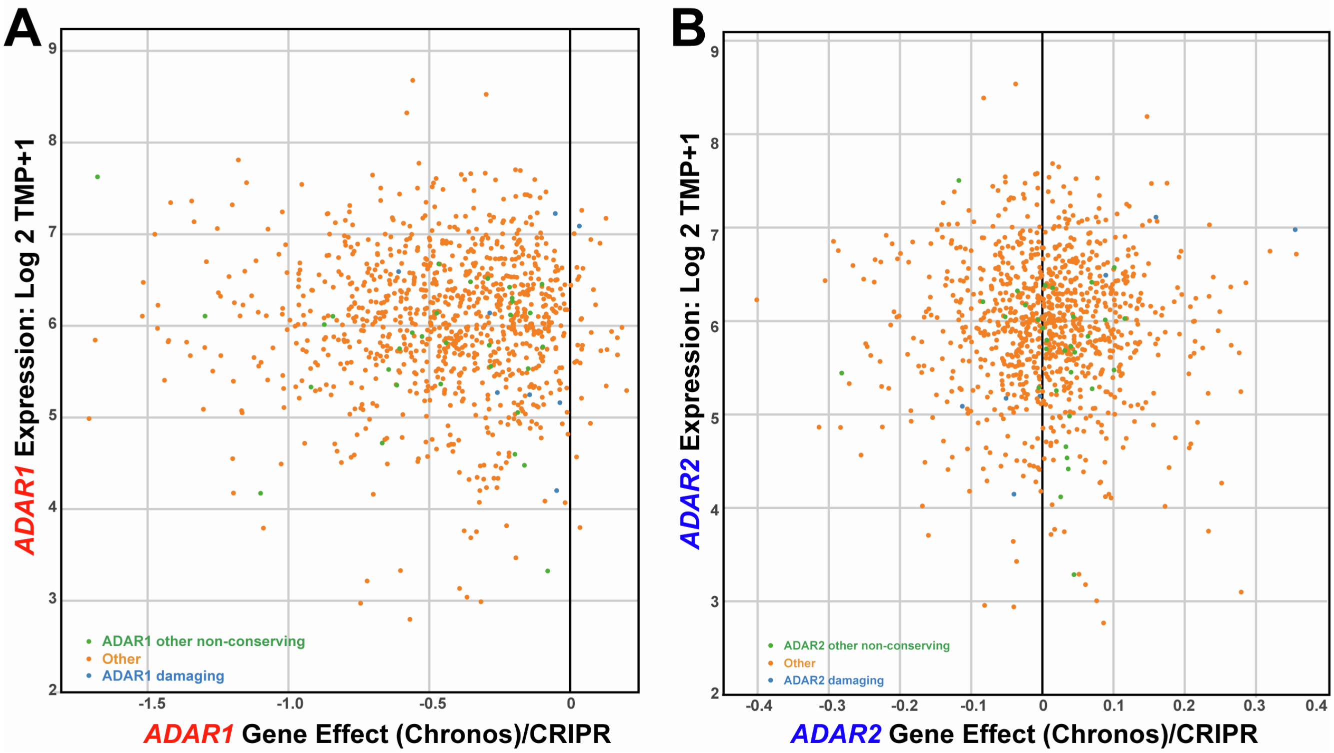

Figure S8. DepMap analysis of ADAR1 and ADAR2, related to Figure 4A

Gene dependency analysis (DepMap) for the survival of human cancer cell lines[44] was conducted at DepMap Portal Site, generated by the Broad Institute (https://doi.org/10.25452/figshare.plus.25880521.v1).

(A) Analysis of ADAR1 dependency in cancer cells by DepMap. The Y axis represents ADAR1 expression levels, while the X axis shows the dependency on ADAR1. Larger minus values indicate a stronger dependency in cancer cell lines. Nearly all human cancer cell lines have developed a dependency on ADAR1.

(B) Analysis of ADAR2 dependency in cancer cells by DepMap. Most cancer cell lines exhibited no dependency on the ADAR2 gene.

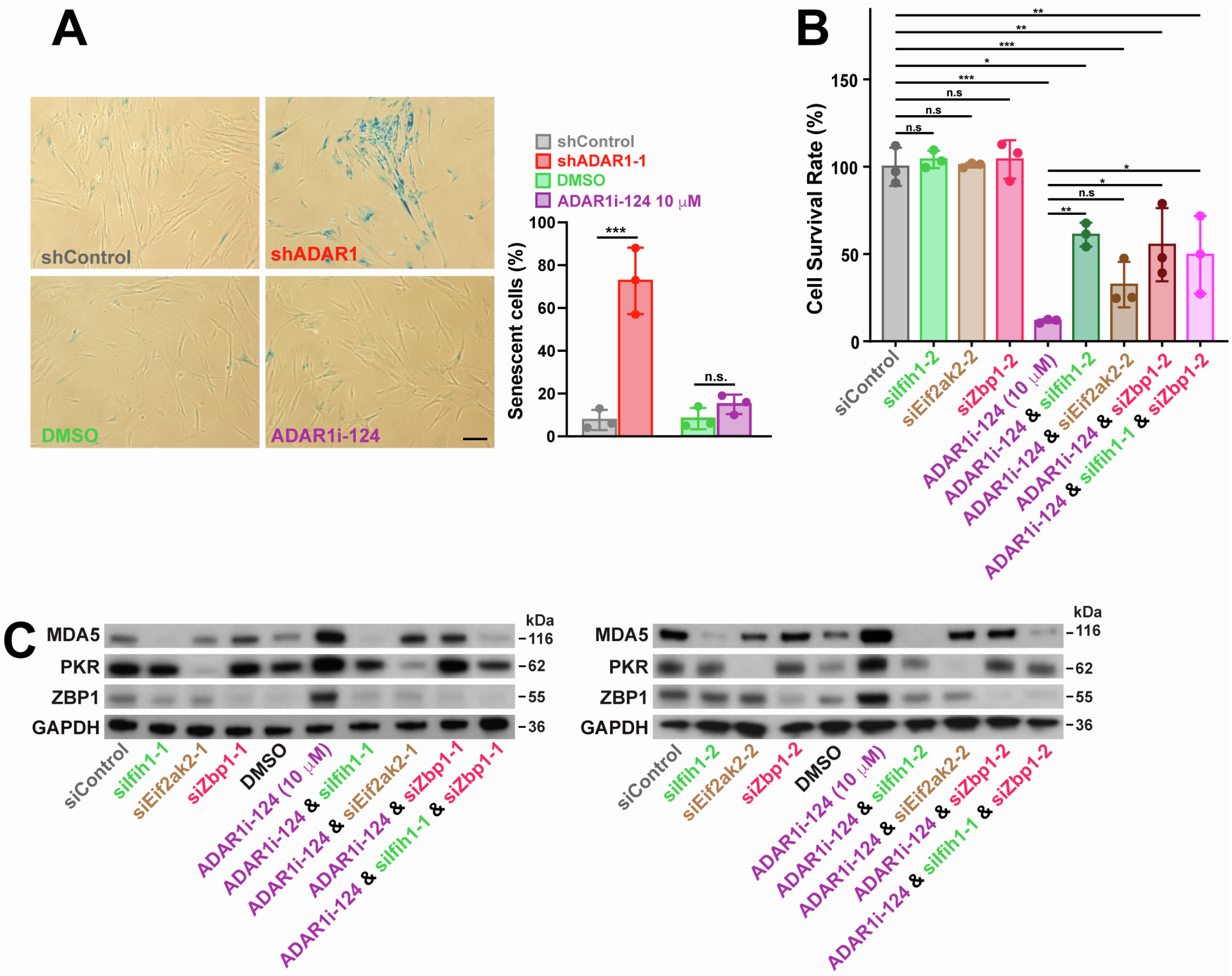

Figure S9. ADAR1i-124 does not inhibit ADAR1 dsRNA binding functions: suppression of cell senescence and PKR activation, related to Figure 4E-F

(A) ADAR1 knockdown, but not ADAR1i-124 treatment, induces cell senescence (as indicated by β-gal staining) in IMR90 fibroblast cells. Scale bar, 50 μm. Data: mean ± SD (n = 3, biological replicates). Significant differences were identified by two-tailed Student's *t*-tests: *n.s.*, not significant; ***P<0.001.

(B) Rescue experiments to counteract the suppression of cell survival rate in ADAR1i-124 treated Yumm1.7 cells were conducted using a second siRNA set (siRNA-2) targeting ADAR1 downstream components. Similar to the results from the first siRNA (siRNA-1) set, ADAR1i-124 mediated inhibition of cell viability was fully reversed by siIfih1-2 (MDA5) and by siZbp1-2, but not by siEif2ak2-2 (PKR). Data: mean ± SD (n = 3 per group, biological replicates). One-way ANOVA followed by Tukey's post hoc test was used to determine the significance. n.s., not significant; *, $P < 0.05$; **, $P < 0.01$; ***, $P < 0.001$.

(C) Efficiency of siRNA knockdown (siRNA-1 and siRNA-2 sets) for three dsRNA sensors, MDA5, PKR, and ZBP1, was confirmed by immunoblotting. GAPDH was used as a loading control. Apparent molecular weights (kDa) are indicated. Full blots with molecular weight markers are shown in Figure S7I: siRNA-1 set and Figure S7J: siRNA-2 set.

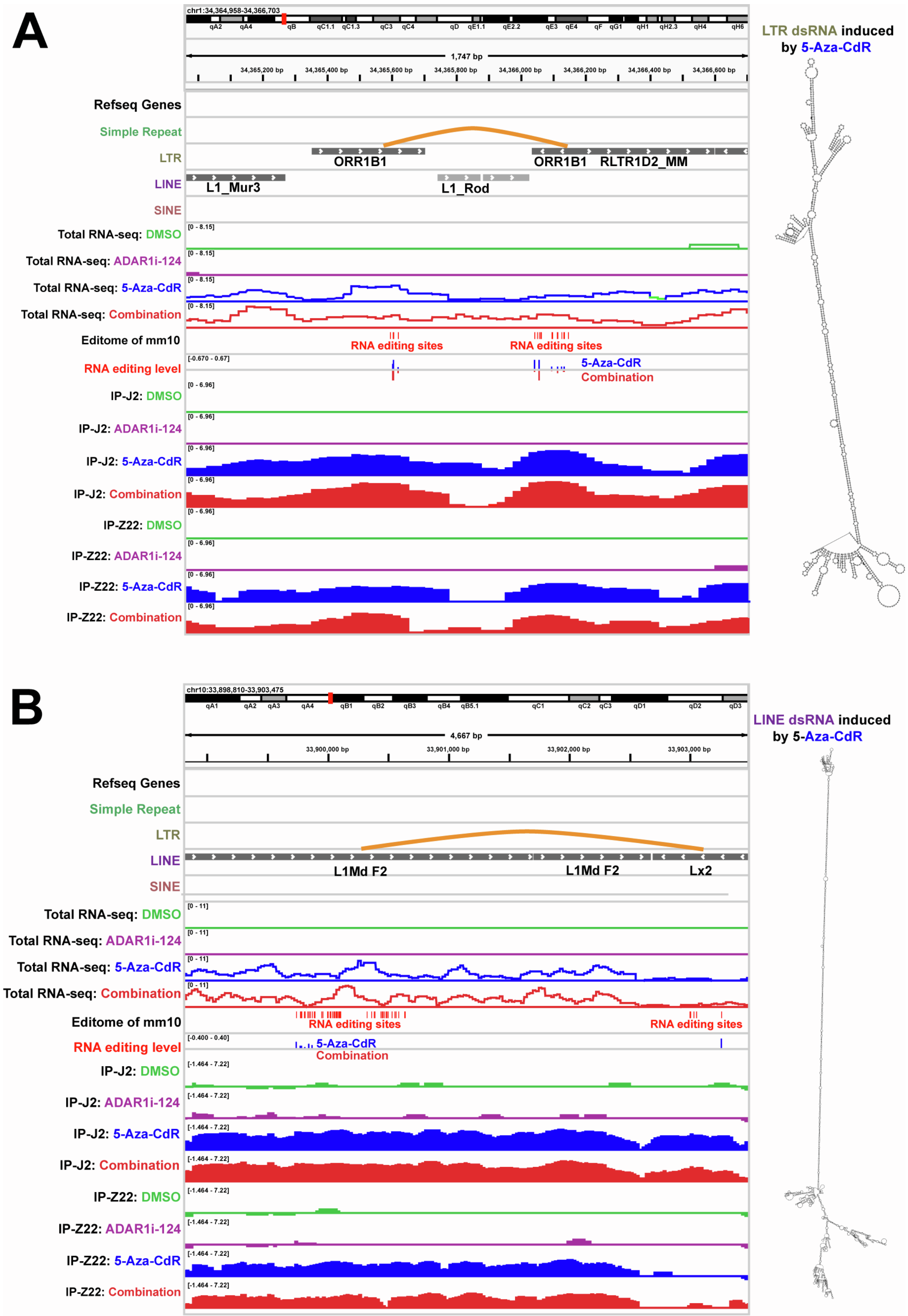

Figure S10. Examples of new dsRNAs induced by 5-Aza-CdR, related to Figure 6

(A) A pair of inverted ERVL LTRs that potentially form dsRNA.

(B) A pair of inverted LINEs that potentially form dsRNA. Both examples are in intergenic regions. The total-RNA-seq tracks depict the expression in RPKM, while the IP tracks indicate the enrichment

(log2ratio) relative to IP-IgG. The dsRNA secondary structures were predicted by RNAfold using the regions containing RNA-editing sites. IR-LTR dsRNA, chr1:34364959-34366703; IR-LINE dsRNA, chr10:33898810-33903475.

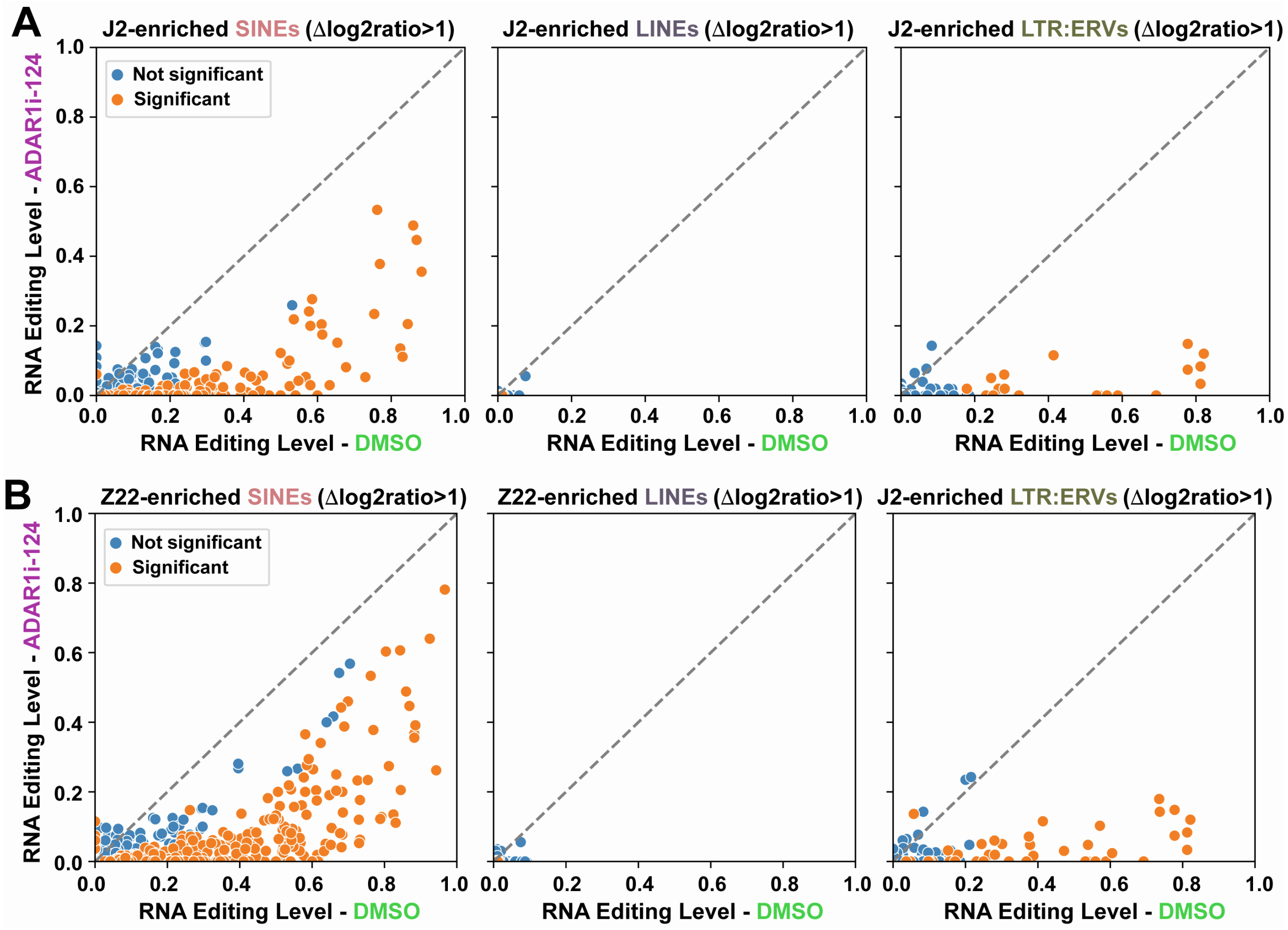

Figure S11. Comparisons of RNA editing levels of sites located on IP-enriched TEs (SINE, LINE, and LTR:ERV subsets), related to Figure 6

(A) Sites on J2-enriched TEs, which is defined as ($log2ratio_{J2,\ ADAR1i\text{-}124}$ - $log2ratio_{J2,\ DMSO}$) > 1 according to Figure 6D.

(B) Sites on Z22-enriched TEs, which is defined as ($log2ratio_{Z22,\ ADAR1i\text{-}124}$ - $log2ratio_{Z22,\ DMSO}$) > 1 according to Figure 6D.

Chi2 tests were used to determine the significance and P<0.05 is considered as significant.

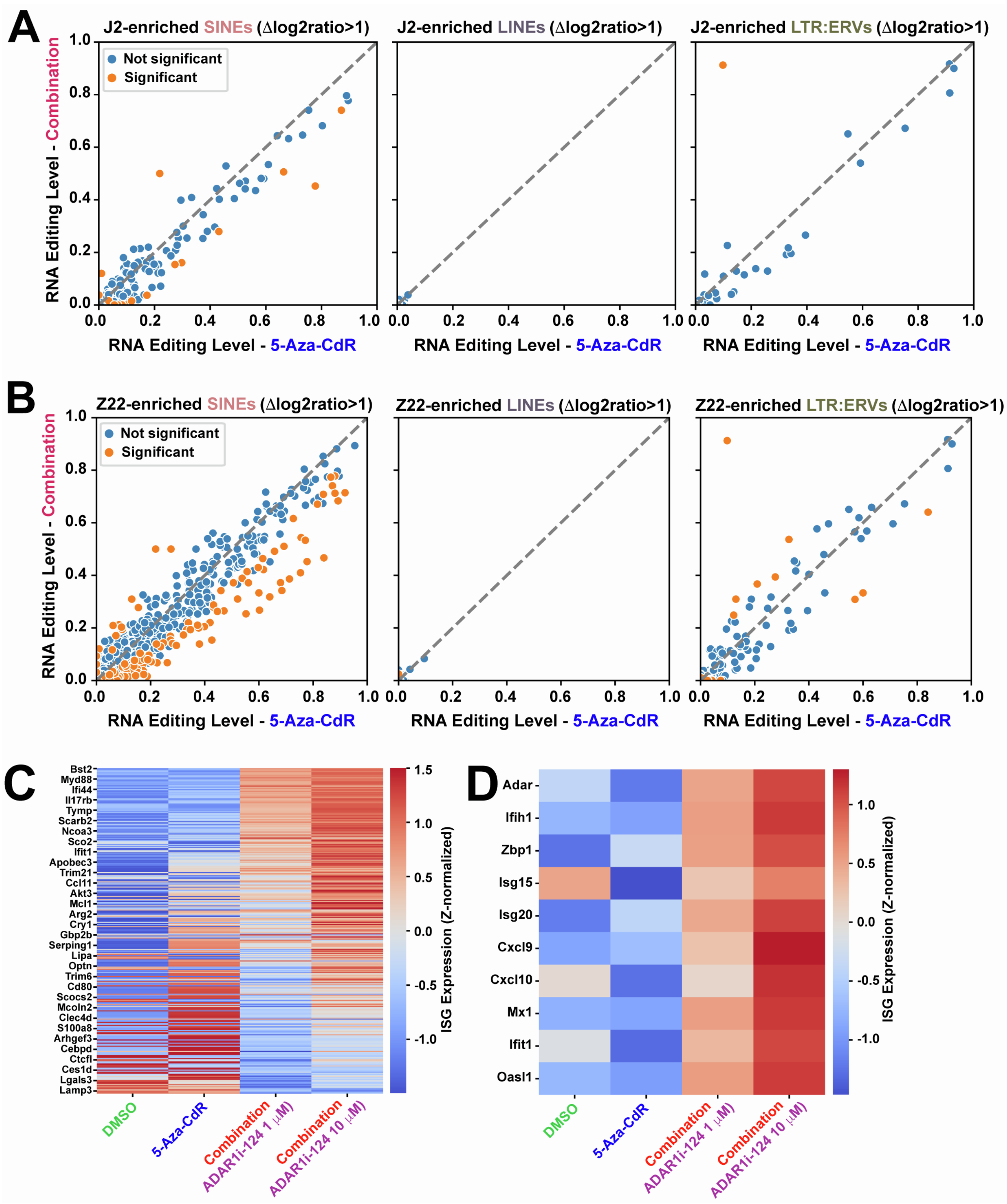

Figure S12. Comparisons of RNA editing levels and expression levels of interferon-stimulated genes (ISGs) between 5-Aza-CdR and combination treatments), related to Figure 6

Yumm1.7 cells were treated with 1 μM 5-Aza-CdR for 3 days and then treated with 10 μM ADAR1i-124 for another 12 hours.

(A) RNA editing levels for sites on J2-enriched TEs, defined as ($log2ratio_{J2,\ ADAR1i\text{-}124}$ - $log2ratio_{J2,\ DMSO}$) > 1.
(B) RNA editing levels for sites on Z22-enriched TEs. The ∆log2ratio threshold and statistical tests are the same as Figure S10. Note that *Adar* (*Adar1*) expression could increase due to strong interferon response at late stages (see below).
(C) The ISGs expressions were obtained from total RNA-seq data and Z-normalized across four groups. Besides 10 μM ADAR1i-124, another dosage (1μM ADAR1i-124) was used to combine with 5-Aza-CdR. At this stage, the type I interferon response was activated in a dose-dependent manner. All 313 mouse ISGs as reported previously[59].
(D) 10 most representative ISGs, including *Adar*. The combinations group of 5-Aza-CdR & 10 μM ADAR1i-124 resulted in the strongest interferon response.

Supplemental Methods

Synthesis of ADAR1i-124

General Methods for Chemistry. Unless otherwise noted, all materials/reagents were obtained from commercial suppliers and used without further purification. Reactions were monitored by liquid chromatography–mass spectrometry (LC-MS) and/or thin layer chromatography (TLC) on silica gel 60 F254 (0.2mm) pre-coated aluminum foil or glass-backed and visualized using UV light. Proton Nuclear Magnetic Resonance (1HNMR, 400 MHz) spectra were recorded on a Bruker NMR spectrometer at room temperature (RT), with TMS or the residual solvent peak as internal standard. The line positions or multiples are given in (δ) and the coupling constants (J) are given as absolute values in Hertz (Hz). The multiplicities in 1HNMR spectra are abbreviated as follows: s (singlet), d (doublet), t (triplet), q (quartet), m (multiplet), br or broad (broadened). Preparative TLC was performed on WhatmanLK6F Silica Gel 60Å size 20x20 cm plates with a thickness of 1000 µm or equivalent. Preparative High Performance Liquid Chromatography (HPLC) purification was performed on Shimadzu LC-6AD. All purification work was completed using Shim-pack PREP-DDS(H)KIT Column. The mobile phases were water (with 0.1% formic acid) and acetonitrile (ACN); all reagents used were HPLC grade. Flow rate was 10 mL/min. LC-MS was performed on a Shimadzu LCMS-2020 equipped with LC-20AD or 30AD pumps, SPD-M20A PDA and Alltech 3300 ELSD. Conditions are as follows: Mobile Phase: A: Water (with 0.1% formic acid), B: ACN; Column: Sepax BR-C18 4.6*50mm, 3µm; Flow Rate: 1.0 mL/min; Oven Temperature: 40 ºC; Gradient: 0.2 min:20% B; 2 min:70% B; 4.8 min:70%B; 5.0 min:20% B; 7.0 min:20% B.

Supplemental Data

Synthetic Scheme for ADAR1i-124

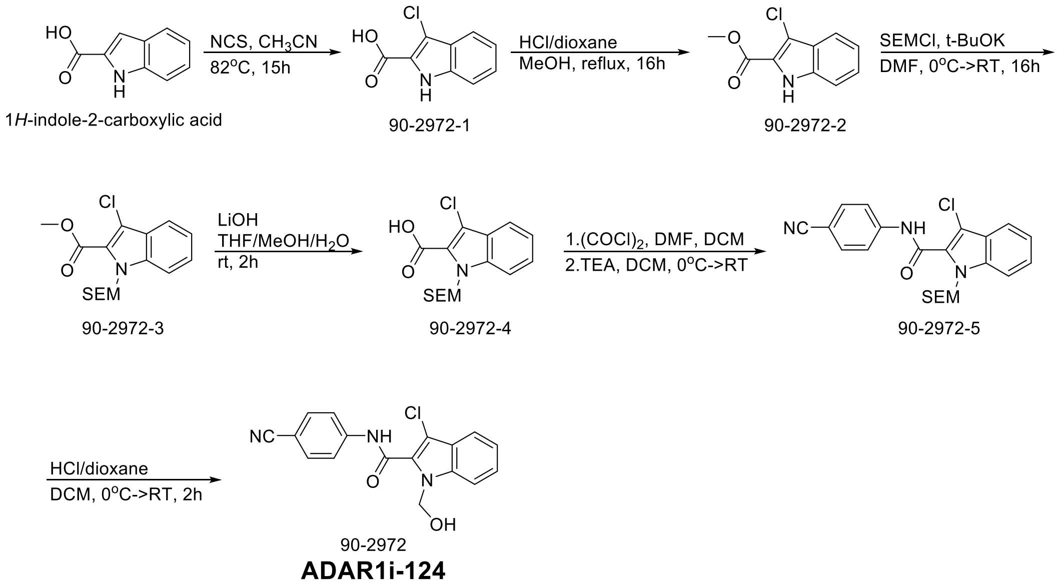

Experimental Details:

Step 1 : Synthesis of 90-2972-1

3-Chloro-1H-indole-2-carboxylic acid

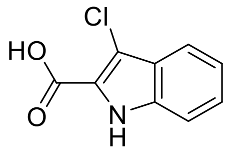

90-2972-1

To a solution of 1H-indole-2-carboxylic acid (5.00 g, 31.03 mmol) in acetonitrile (15 mL) at room temperature was added N-Chlorosuccinimide (4.55 g, 34.13 mmol). The reaction mixture was stirred at 82oC under nitrogen atmosphere overnight. Afterward, the mixture was cooled to room temperature and concentrated. The residue was then treated with water (30 mL) and stirred at room temperature for 1 hour. The resulting solid was collected by filtration and dried. 6.00 g 3-chloro-1H-indole-2-carboxylic acid (90-2972-1) was obtained (98.8% yield, 96.5% purity) as a brown solid. LC_MS: 195.80 [M+H]+. tR = 2.560 min.
1HNMR (400 MHz, DMSO-d6): δ 13.58-13.31 (m, 1H), 12.04 (s, 1H), 7.60 (d, J = 8.0 Hz, 1H), 7.47 (d, J = 8.4 Hz, 1H), 7.37-7.33 (m, 1H), 7.18 (d, J = 8.0 Hz, 1H).
Chemical Formula: C9H6ClNO2, Molecular Weight: 195.60.

Step 2: Synthesis of 90-2972-2
Methyl 3-chloro-1H-indole-2-carboxylate

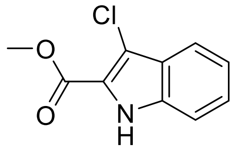

90-2972-2

A mixture of 3-chloro-1H-indole-2-carboxylic acid (90-2972-1, 6.0 g, 30.67 mmol) and a 4.0 M HCl-dioxane solution (50 mL) in methanol (100 mL) was refluxed for 16 hours. The solution was then concentrated under vacuum. The residue was diluted with ethyl acetate (50 mL) and carefully alkalinized with saturated aqueous sodium bicarbonate solution (100 mL). The organic layer was separated, and the aqueous phase was extracted with ethyl acetate (50 mL × 2). The combined organic layers were washed with brine (50 mL), dried over sodium sulfate, filtered, and concentrated under reduced pressure. The resulting product, methyl 3-chloro-1H-indole-2-carboxylate (90-2972-2), was obtained as a brown solid (5.25 g, 81.6% yield, 95.2% purity).
LC_MS: 209.80 [M+H]+. tR = 3.034 min.
1H NMR (400 MHz, DMSO-d6): δ 12.20 (s, 1H), 7.62 (d, J = 8.0 Hz, 1H), 7.49 (d, J = 8.4 Hz, 1H), 7.39-7.35 (m, 1H), 7.22-7.18 (m, 1H), 3.92 (s, 3H).
Chemical Formula: C10H8ClNO2, Molecular Weight: 209.63.

Step 3: Synthesis of 90-2972-3
Methyl 3-chloro-1-((2-(trimethylsilyl) ethoxy) methyl)-1H-indole-2-carboxylate

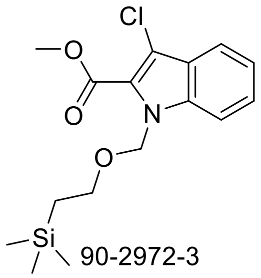

90-2972-3

To a stirred mixture of methyl 3-chloro-1H-indole-2-carboxylate (90-2972-2, 5.25 g, 25.04 mmol) in dimethylformamide (DMF, 55 mL) at 0°C under a nitrogen atmosphere, potassium tert-butoxide (2.95 g, 26.30 mmol) was added. The mixture was stirred at 0–5°C for 30 minutes, followed by the dropwise addition of 2-(trimethylsilyl) ethoxymethyl chloride (4.59 g, 27.55 mmol). The reaction mixture was then stirred at room temperature for 16 hours. To quench the reaction, water (120 mL) was added, and the mixture was extracted with ethyl acetate (150 mL). The organic phase was washed with brine (100 mL), dried over sodium sulfate, and concentrated. The crude product was purified by silica gel flash chromatography (eluted with PE/EtOAc, 40:1 V/V) to yield 3-chloro-1-((2-(trimethylsilyl) ethoxy) methyl)-1H-indole-2-carboxylate (90-2972-3, 7.4 g, 87.0% yield, 97.7% purity) as a colorless oil.
1HNMR (400 MHz, CDCl3): δ 7.73 (d, J = 8.0 Hz, 1H), 7.55 (d, J = 8.4 Hz, 1H), 7.44-7.40 (m, 1H), 7.28-7.24 (m, 1H), 5.95 (s, 2H), 3.99 (s, 3H), 3.50 (t, J = 8.0 Hz, 2H), 0.86 (t, J = 8.0 Hz, 2H), -0.08 (s, 9H).
Chemical Formula: C16H22ClNO3Si, Molecular Weight: 339.89.

Step 4: Synthesis of 90-2972-4
3-Chloro-1-((2-(trimethylsilyl)ethoxy)methyl)-1H-indole-2-carboxylic acid

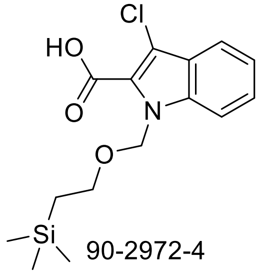

90-2972-4

A solution of methyl 3-chloro-1-((2-(trimethylsilyl) ethoxy) methyl)-1H-indole-2-carboxylate (90-2972-4, 7.4 g, 21.77 mmol) and lithium hydroxide (3.66 g, 87.09 mmol) in a mixture of tetrahydrofuran (30 mL), methanol (30 mL), and water (15 mL) was stirred at room temperature for 2 hours. The reaction mixture was then cooled to 0°C, and the pH was adjusted to 3-4 using 2N HCl. The mixture was extracted with ethyl acetate (80 mL × 2). The combined organic layers were dried over sodium sulfate and concentrated under vacuum. The residue was purified by silica gel flash chromatography (eluted with DCM/MeOH, 30:1 V/V), yielding 3-chloro-1-((2-(trimethylsilyl) ethoxy) methyl)-1H-indole-2-carboxylic acid (90-2972-4) as a colorless oil (5.0 g, 70.4% yield, 98.1% purity).
1H NMR (400 MHz, CDCl3) δ 7.76 (d, J = 8.0 Hz, 1H), 7.57 (d, J = 8.4 Hz, 1H), 7.48-7.44 (m, 1H), 7.30-7.28 (m, 1H), 5.99 (s, 2H), 3.55 (t, J = 8.4 Hz, 2H), 0.88 (t, J = 8.0 Hz, 2H), -0.07 (s, 9H).
Chemical Formula: C15H20ClNO3Si, Molecular Weight: 325.86.

Step 5: Synthesis of 90-2972-5
3-Chloro-N-(4-cyanophenyl)-1-((2-(trimethylsilyl)ethoxy)methyl)-1H-indole-2-carboxamide

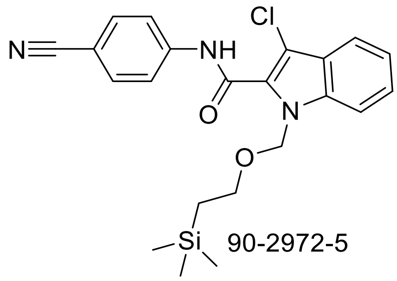

To a mixture of 3-chloro-1-((2-(trimethylsilyl) ethoxy) methyl)-1H-indole-2-carboxylic acid (90-2972-4, 5.0 g, 15.34 mmol) and a catalytic amount of dimethylformamide (0.5 mg) in dichloromethane (45 mL) at 0-5°C under a nitrogen atmosphere, oxalyl chloride (7.79 g, 61.38 mmol) was added. The mixture was stirred at room temperature for 1 hour. Afterward, the mixture was concentrated, and the residue was re-dissolved in dichloromethane (10 mL), forming a solution. This solution was then added to a mixture of 4-aminobenzene-1-carbonitrile (1.81 g, 15.34 mmol) and triethylamine (3.10 g, 30.69 mmol) in dichloromethane (10 mL). The resulting mixture was stirred at room temperature for 2 hours. The solution was concentrated under vacuum and diluted with water (80 mL). The mixture was extracted with ethyl acetate (100 mL), washed with brine (50 mL), dried over sodium sulfate, and concentrated. The crude product was purified by silica gel flash chromatography (eluted with PE/EtOAc, from 15:1 to 10:1 V/V) to yield 3-chloro-N-(4-cyanophenyl)-1-((2-(trimethylsilyl) ethoxy) methyl)-1H-indole-2-carboxamide (90-2972-5) as a white solid (3.1 g, 47.7% yield, 98.3% purity).
1H NMR (400 MHz, CDCl3) δ 8.94 (s, 1H), 7.83 (d, J = 8.8 Hz, 2H), 7.32-7.67 (m, 3H), 7.57 (d, J = 8.4 Hz, 1H), 7.48-7.44 (m, 1H), 7.31 (t, J = 7.6 Hz, 1H), 5.96 (s, 2H), 3.61 (t, J = 8.0 Hz, 2H), 0.91 (t, J = 8.4 Hz, 2H), -0.08 (s, 9H).
Chemical Formula: C22H24ClN3O2Si, Molecular Weight: 425.99.

Step 6: Synthesis of 90-2972; **ADAR1i-124**
3-Chloro-N-(4-cyanophenyl)-1-(hydroxymethyl)-1H-indole-2-carboxamide

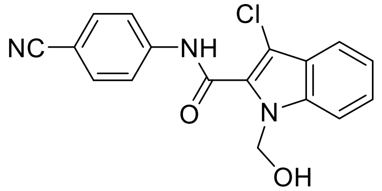

90-2972
ADAR1i-124

To a solution of 3-chloro-N-(4-cyanophenyl)-1-((2-(trimethylsilyl) ethoxy) methyl)-1H-indole-2-carboxamide (90-2972-5, 3.8 g, 8.92 mmol) in dichloromethane at 0-5°C, HCl in dioxane (4.0 M, 38 mL) was added dropwise. The mixture was stirred at room temperature for 2 hours. The resulting solution was concentrated under vacuum and recrystallized from a 1:1 mixture of n-hexane and ethyl acetate. The product, 3-chloro-N-(4-cyanophenyl)-1-(hydroxymethyl)-1H-indole-2-carboxamide (90-2972), was obtained as a white solid (1.38 g, 47.5% yield, 97.5% purity).
1H NMR (400 MHz, CDCl3) δ 8.80 (s, 1H), 7.84 (d, J = 8.8 Hz, 2H), 7.75-7.69 (m, 3H), 7.58-7.53 (m, 2H), 7.41-7.37 (m, 1H), 6.59 (s, 2H),
Chemical Formula: C17H12ClN3O2, Molecular Weight: 325.75.
